# A Next-Generation Vaccine Candidate Using Alternative Epitopes to Protect against Wuhan and All Significant Mutant Variants of SARS-CoV-2: An Immunoinformatics Approach

**DOI:** 10.14336/AD.2021.0518

**Published:** 2021-12-01

**Authors:** Manojit Bhattacharya, Ashish Ranjan Sharma, Pratik Ghosh, Sang-Soo Lee, Chiranjib Chakraborty

**Affiliations:** ^1^Department of Zoology, Fakir Mohan University, Vyasa Vihar, Balasore-756020, Odisha, India.; ^2^Institute for Skeletal Aging & Orthopedic Surgery, Hallym University-Chuncheon Sacred Heart Hospital, Chuncheon-si, 24252, Gangwon-do, Republic of Korea.; ^3^Department of Zoology, Vidyasagar University, Midnapore, West Bengal 721102, India.; ^4^Department of Biotechnology, School of Life Science and Biotechnology, Adamas University, Barasat-Barrackpore Rd, Kolkata, West Bengal 700126, India.

**Keywords:** SARS-CoV-2, mutant variants, next-generation vaccine, peptide vaccine, multi-epitopes

## Abstract

Newly emerging significant SARS-CoV-2 variants such as B.1.1.7, B.1.351, and B.1.1.28 are the variant of concern (VOC) for the human race. These variants are getting challenging to contain from spreading worldwide. Because of these variants, the second wave has started in various countries and is threatening human civilization. Thus, we require efficient vaccines that can combat all emerging variants of SARS-CoV-2. Therefore, we took the initiative to develop a peptide-based next-generation vaccine using four variants (Wuhan variant, B.1.1.7, B.1.351, and B.1.1.28) that could potentially combat SARS-CoV-2 variants. We applied a series of computational tools, servers, and software to identify the most significant epitopes present on the mutagenic regions of SARS-CoV-2 variants. The immunoinformatics approaches were used to identify common B cell derived T cell epitopes, influencing the host immune system. Consequently, to develop a novel vaccine candidate, the antigenic epitopes were linked with a flexible and stable peptide linker, and the adjuvant was added at the N-terminal end. 3D vaccine candidate structure was refined, and quality was assessed using web servers. The physicochemical properties and safety parameters of the vaccine construct were assessed through bioinformatics and immunoinformatics tools. The molecular docking analysis between TLR4/MD2 and the proposed vaccine candidate demonstrated a satisfactory interaction. The molecular dynamics studies confirmed the stability of the vaccine candidate. Finally, we optimized the proposed vaccine through codon optimization and *in silico* cloning to study the expression. Our multi-epitopic next-generation peptide vaccine construct can boost immunity against the Wuhan variant and all significant mutant variants of SARS-CoV-2.

On March 11, 2020, WHO announced the recent novel coronavirus (SARS-CoV-2) outburst as a pandemic [1-3]. To date, this outbreak has killed millions of people and resulted in consequential economic losses worldwide. The SARS-CoV-2 belongs to the β coronaviruses family and is nearly associated with SARS-CoV and MERS-CoV, responsible for the outbreak in 2002 and 2012 correspondingly [4-8]. In November-December 2020, after the gradual weakening of the first pandemic wave, COVID-19 was under control within many large countries like India, China, Australia, etc. However, Brazil, USA, and Mexico were not like other countries and suffered during the above-mentioned period [9]. Currently, the COVID-19 crisis is much worse in India, Italy, Brazil, and Mexico. The second COVID-19 surge penetrated all over the countries and has recorded the highest daily infection and death [10]. This situation arose due to the emergence of new SARS-CoV-2 variants.

As per the latest research reports on COVID-19, three newly identified variants were assigned to lineages 501Y.V1 (B.1.1.7), 501Y.V2 (B.1.351), and P.1 (B.1.1.28) are very infective. Galloway et al. reported SARS-CoV-2 variant lineage B.1.1.7 as a significant cause of concern in the United Kingdom (UK) [11,12]. The 501Y.V2 or B.1.351 lineage first emerged in the Nelson Mandela Bay area of Eastern Cape Province, South Africa, and different locations in Eastern and Western Cape Provinces [9, 13]. Another important SARS-CoV-2 variant with 17 unique mutations was reported from Brazil. In this variant, one out of ten mutations is in spike glycoprotein. Among them, mutations that have been designated to be of particular concern are N501Y, K417T, and E484K [14]. This lineage is called as P.1 (B.1.1.28) lineage, also known as 20J/501Y.V3, Variant of Concern (VOC) 202101/02. The B.1.1.28 circulating in Manaus, capital of Amazonas, Brazil spread to five other countries, including Germany, Italy, Japan, and South Korea [15-18].

The current scenario indicates the crucial non-synonymous (ns) or point mutations and deletions occurring in the emerging SARS-CoV-2 variants, i.e., 501Y.V1, 501Y.V2, and P.1 variants and their possible impact on the overall structure-function of SARS-CoV-2 proteins. Therefore, the structural changes in spike glycoprotein (S-protein) through mutation impact viral functions and harms immunization programs. Furthermore, the mutant variants affect the neutralizing antibodies; hence a modified next-generation vaccine candidate using alternative epitopes is urgently required against these three threatening variants and the Wuhan variant.

**Figure 1.**
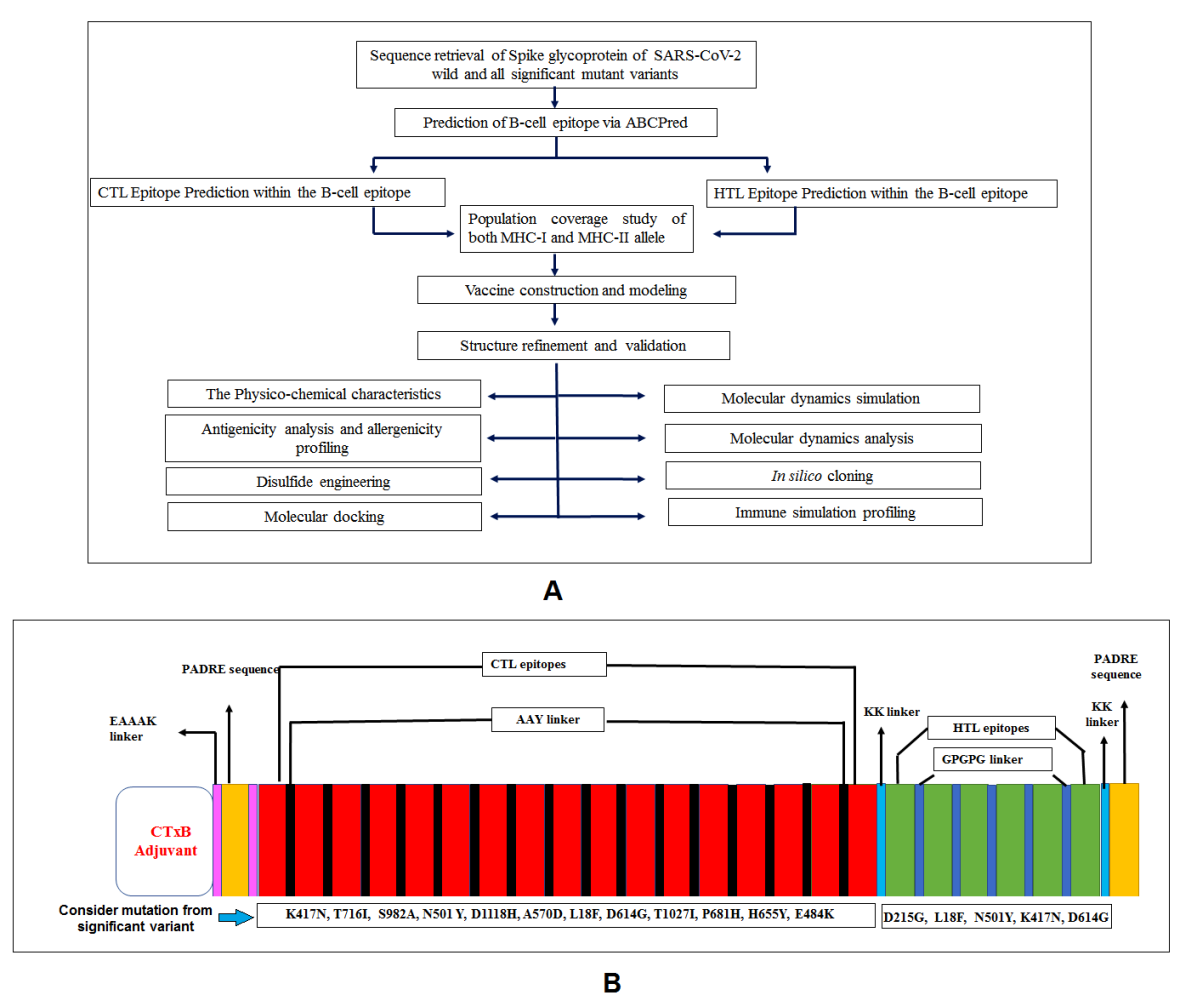
Graphical representation of standard methodology and variants mutation. A) Flow chart of the adopted methodology for designing of next-generation epitopic vaccine. B) Our vaccine construct illustrates all significant mutation presents in selected epitopes from major variants of SARS-CoV-2.

This study aims to design a multi-epitope peptide-based vaccine using alternative epitopes of these four variants that could efficiently elicit both innate and adaptive immune responses. Our vaccine development strategies using immunoinformatics and bioinformatics approaches are fast, precise, cost-effective, and reliable against pathogens [19, 20]. Therefore, we carried out several immunoinformatics studies to identify B cell, Cytotoxic T lymphocytes (CTL), and Helper T lymphocyte (HTL) epitopes from the S proteins of all four variants (Wuhan variant and other three newly emerged variants). We also combined adjuvants and PADRE sequence at the N-terminal of protein vaccine candidate having the most promising common B cell and T cell (CTL and HTL) epitope along with suitable peptide linker. Furthermore, we have calculated the vaccine candidate’s antigenicity and physicochemical properties through bioinformatics and immunoinformatics approaches. The modeled 3D vaccine structure was docked against the TLR4/MD2 complex to ensure its efficacy of inducing an immune response [21]. Additionally, the docked complex was validated and analyzed with molecular dynamics (MD) simulations. By using immune simulation profiling, we also assessed the vaccine candidate’s immune response on its administration to the human body and calculated the extent of elevation of primary and secondary immunoglobulin. Subsequently, we performed *in silico* cloning to ensure the expression and translation efficiency of the proposed vaccine candidate. Our study tried to generate a potential and effective vaccine candidate against the emerging SARS-CoV-2 variants and provide a platform for future researchers to develop a suitable next-generation peptide-based vaccine against the Wuhan and all significant mutant variants of this virus to resist the COVID-19 pandemic.

## MATERIAL AND METHODS

The standard methodologies applied to execute existing work are listed in a flow chart (Fig. 1A). Every step of this adopted methodology is crucial and promising for the successful design of the desired multi-epitopic peptide-based vaccine against SARS-CoV-2 Wuhan and all significant mutant variants (Fig. 1B).

We have taken a positive control (C1) for the validation of the primary sequence of the vaccine construct and verify the antigenicity, physicochemical properties, molecular docking, and immune simulation. We utilized the multi-epitope peptide-based vaccine candidate composed of antigenic epitopes of SARS-CoV-2 S-protein and Orf1ab polyprotein antigens designed by Safavi et al., 2020 as a positive control (C1) [22].

### Retrieval of the protein sequence

Amino acid sequences of the S-protein of three newly emerging SARS-CoV-2 mutant lineage and the Wuhan variant were retrieved from the NCBI protein database in FASTA format [23]; their accession numbers are tabulated in Table 1.

**Table 1:** SARS-CoV-2 Wuhan variant and newly emerge mutative variants name and their significant mutation.

Variant name	NCBI accession number	Numbers of amino acid	Lineage	Important mutation	Other name	Virulence
Wuhan variant	QHR63290.2	1273	-	-	-	High
United Kingdom variant	7LWV_A	1285	B.1.1.7	N501Y, 69-70 and 144 del, P681H, D614G, D1118H, A570D, T716I, S982A, T1027I	501Y.V1	Severe
South Africa variant	7LYL_A	1288	B.1.351	N501Y, K417N, E484K, D80A, D215G, A701V, L18F,	501Y.V2	Low
Brazil variant	7LWW_A	1288	P.1/P.2	N501Y, E484K, K417T, H655Y	B.1.1.28	Severe

### Identification of the B cell epitopes

The ABCpred webserver was used to predict B cell epitope(s) in an antigen sequence-based artificial neural network [24]. The server is based on a recurrent neural network (machine-based technique) to predict linear B cell epitopes [26]. We have used 20 window lengths for B cell epitope identification, as indicated epitope lengths in supplementary file-1.

### Prediction and identification of CTL and HTL epitopes

IEDB recommended 2020.09 (NetMHCpan EL 4.1), and IEDB recommended 2.22 was employed to predict CTL and HTL epitopes within the studied S-proteins [26, 27].

### Cluster analysis of the MHC alleles

Cluster analysis was achieved using the MHCcluster 2.0 online server [28]. Cluster analysis of the MHC alleles predicts the alleles of the MHC class-I and class-II molecules with similar binding patterns. During cluster analysis, the default parameter of the server was kept as follows: the number of peptides at 50000, the number of bootstraps at 100, and the fraction of peptides to include the correlation analysis at 10. Here the servers used NetMHCpan-2.8 and NetMHCpan-2.8 prediction methods for allele cluster analysis.

### Determination of population coverage

We have determined the large number of HLA binding antigenic epitope peptides, which provides the information of the population coverage through its interaction pattern. The IEDB tool assisted in predicting the population coverage of the potent epitope interacts with the HLA allele (MHC-II restriction based) of a particular population coverage [29, 30].

### Construction of multi-epitopic vaccine candidate

**Figure 2.**
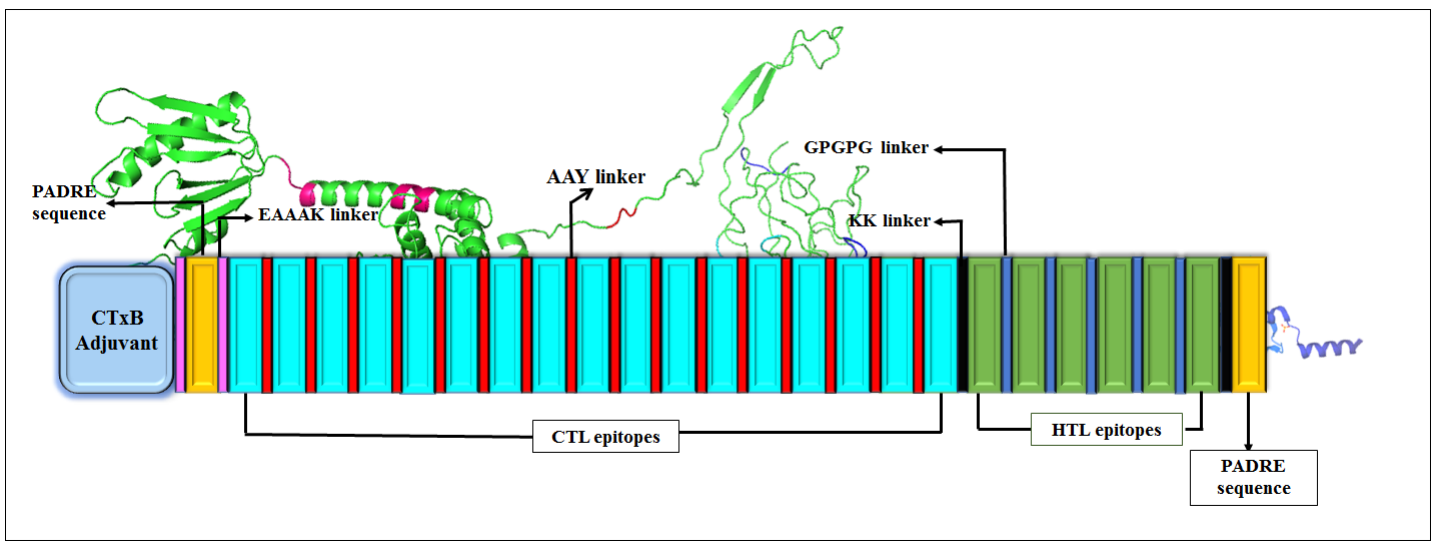
Graphical representation of final vaccine construct from epitopes with different peptide linkers.

The final vaccine candidate was constructed by joining common B cell derivate CTL and HTL epitopes. Linkers such as AAY and GPGPG were taken to fuse CTL and HTL epitopes, correspondingly. Linker plays a crucial role in improving the separation of epitopes, and it also assists epitopes presentation towards MHC class I, II receptors, and immune processing [31, 32]. Furthermore, to boost the immunity, CTxB adjuvant was attached to the N-terminal of the chimeric sequences by an EAAAK linker to the vaccine candidate. CTxB is the nontoxic segment (124 amino acids long) of cholera toxin that can provoke cellular and humoral immune responses [33]. Finally, an additional PADRE sequence (AKFVAA WTLKAAA) with 13 amino acids was added at C-terminal to improve immunogenicity within the host body [34]. The graphical representation of the constructed vaccine is shown in Figure 2.

### Allergenicity, primary and secondary structure analysis

The allergenicity of the vaccine was analyzed by the automated web server, namely AllerTop 2.0 and AllergenFP v.1.0 [35,36]. These servers are based on the Auto Cross Covariance (ACC) method for transforming protein sequences into uniform equal-length vectors.

The primary structure of the vaccine candidate was assessed through Expasy ProtParam tool [37]. This server computes a set of physical and chemical characteristics of the input sequence, such as the number of amino acids, molecular weight, isoelectric point (pI) where the protein sequence has no net charges, grand average of hydropathicity (GRAVY) instability index (II), aliphatic index, the number of total atoms, and estimated half-life of the interest peptide sequence. To further characterize different properties of our vaccine construct, we considered an established multi-epitopic vaccine candidate as a positive control. For allergenicity evaluation, we have used a positive control.

The secondary structure was analyzed using the self-optimized prediction method of SOPMA [38] and PSIPRED 4.0 [39]. These tools allow access to properties like transmembrane helices, globular regions, bend regions, random coil, and coiled-coil regions to obtain a graphical presentation of the protein sequence of interest.

**Figure 3.**
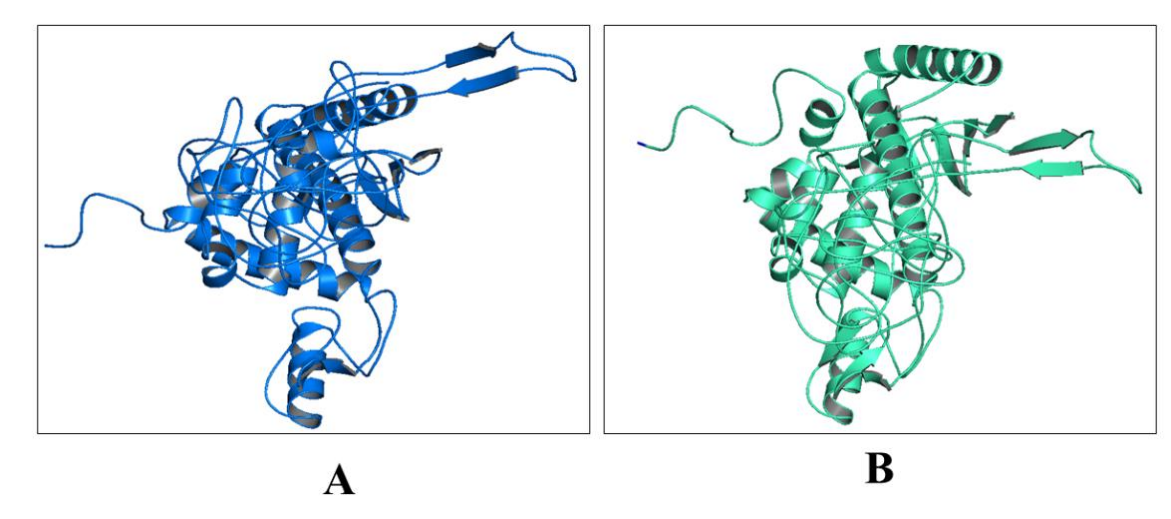
3D model of vaccine construct. A) Naïve structure, B) Refined structure.

### Prediction of antigenicity

The antigenicity of our construct was analyzed along with positive control (C1) using the VaxiJen webserver. This server use a new alignment-independent method for antigen prediction based on ACC transformation of peptide sequences [40]. It was also noted that the VaxiJen is a reliable and consistent server for predicting protective antigens, which has a default threshold value (0.4%) for predicting antigenicity of viruses.

### Three-dimensional (3D) modeling of vaccine and model refinement

3D structure of the vaccine construct was developed by using the Raptor-x server [41, 42]. 3D protein architectures provide crucial information for an atomic-level understanding of molecular functions. This is a template-based protein structure modeling server; that generates a good quality model with proper coordinate formation (Fig. 3A). The GalaxyWEB server was used for 3D model refinement [43]. The refined model is represented in the Figure 3B.

### Validation of peptide-based vaccine structure

Validation of the predicted 3D model of the vaccine structure is a significant step, and mainly three web-based servers were assigned to validate the protein model, such as ProSA, ERRAT, and PROCHECK. The ProSA is an online server that is used to validate the tertiary structure of the protein [44]. The overall quality scoring was computed by the local quality model and “Z’ plot. The ERRAT server was used for statistical analysis of the non-bonded interaction of the model protein [45]. Finally, we used the PROCHECK server for Ramachandran plot analysis [46].

### Disulfide engineering of the 3D multi-epitope vaccine construct

Disulfide by Design 2 (DbD2) tool was used for increased protein stability as well as decreased conformational entropy [47]; this calculation is a measurement of uncertainty or randomness by the addition of novel disulfide bonds, and it changes the 3D conformations of the modeled vaccine construct. Here, we employed the DbD2 web tool for disulfide engineering of the 3D structured multi-epitope vaccine construct that somehow might increase the protein-protein interactions.

### Molecular docking of vaccine constructs with TLR4/MD2 complex

The TLR4/MD2 complex has a vital role in eliciting immune responses against infection by microbes. The binding interactions of antigenic vaccine-candidate with the target immune cell protein are crucial for creating a suitable immune response. For calculating the binding interaction of the vaccine candidate with TLR4/MD2 complex (PDB ID: 3FXI) we applied the PyMoL stand-alone tool to remove unnecessary items from the PDB coordinates [48], molecular docking analysis was performed by HDOCK and HawkDock online servers [49,50]. Here, we used the C1 construct as positive control and assessed molecular docking interaction with our designed vaccine candidate. Interaction of C1 with TLR4 was used as a positive control to compare the interaction property of our vaccine construct and TLR4/MD2 complex.

Furthermore, the HawkDock server calculates free binding energy with the base of MM/GBSA free energy calculating mechanisms. Therefore, this molecular complex was chosen as the primary structure for initiating the MD simulation.

**Figure 4.**
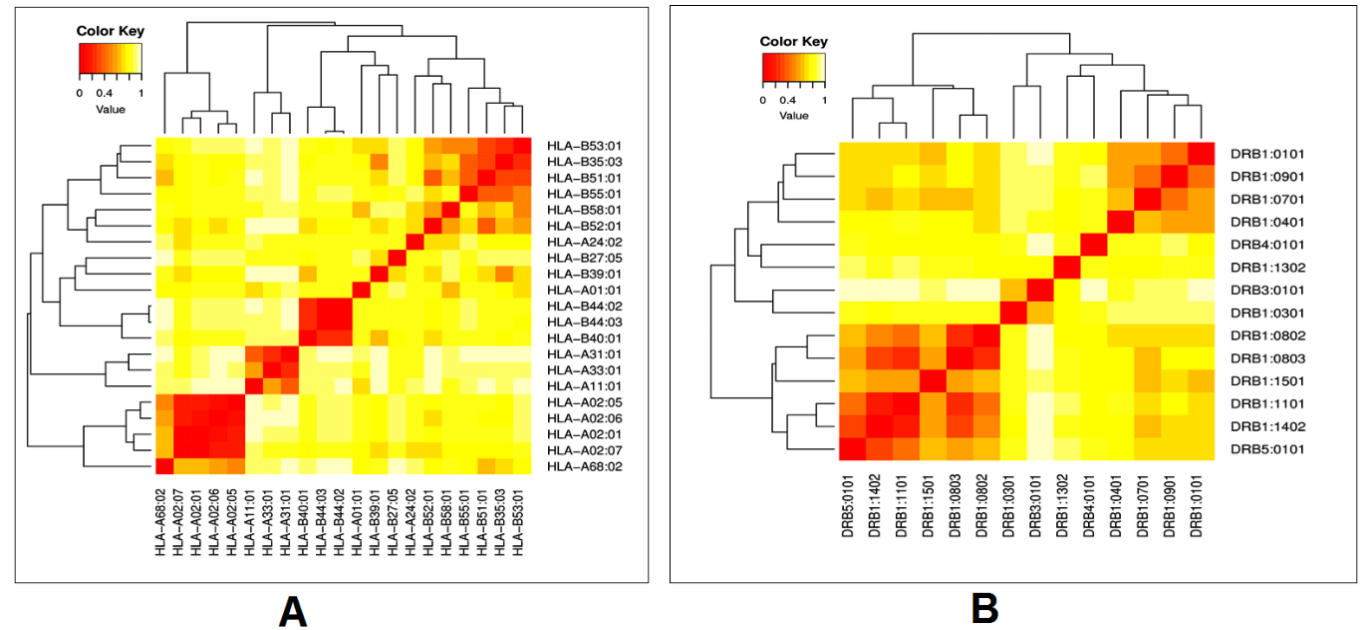
Cluster analysis of the MHC HLA alleles where A) is the clustering of MHC-I HLA alleles and B) is the clustering of the MHC-II HLA alleles (red colors is the indication of the strong interaction, while the yellow zone indicates weaker interaction).

### Molecular dynamics simulation

Molecular dynamics (MD) simulations were carried out using Nanoscale Molecular Dynamics (NAMD), a molecular dynamics program designed for high-performance simulation of large biomolecular systems [51]. Protein topologies were prepared by the psf module of NAMD using the CHARMM22 and CHARM36 all-atom force field for protein molecules and the TIP3P (point) water (W) was added as solvation [52]. A cube box was defined using the protein with a position of at least 0.4 nm from the box edge. We added 11 sodium ions and 0 CL ions to neutralize the overall charge of the solvated protein structure. The energy was minimized through NAMD energy minimizer with a maximum 1,000 steps of the steepest descent minimization algorithm. In the preparation of NVT restrained phases, the temperature was set at 310K at a fixed pressure, and then the same pressure was used in NPT (isothermal-isobaric) ensemble. Finally, we achieved unrestrained (NVT and NPT free) MD simulations of the equilibrated systems and ran MD simulations with 20ns (time step) with 0.2fs speed. We analyzed simulation trajectories files using VMD and analyzed some other secondary analysis types like bond angle, dihedral, etc. [53].

### Normal mode analysis

The iMODS online webserver was used to illustrate the collective motion of peptide vaccine through normal modes (NMA) in internal coordinates [54]. Essential dynamics simulation (EDM) is a commanding approach and helps to find out the macromolecular mobility and stability. This webserver predicted the immanent motions of the complex in terms of deformability, B-factors, eigenvalues, covariance, and elastic model.

### Immune simulation profiling

The C-ImmSim web-based server was applied to characterize the comparative analysis of immune response profiling of our designed vaccine candidate and positive control (C1) without any adjuvant [55]. This server describes both the humoral and cellular response of a mammalian immune system to the presence of immunogens or antigens such as viruses, bacteria, etc., at the cellular level (mesoscopic scale). The agent-based server was used to predict antigenic immune response by the position-specific scoring matrix (PSSM) and machine learning approaches. In this analysis, several parameters such as random seed, simulation volume, and simulation step were customized at 12345, 25, and 1000, respectively. In this model, we applied two injections with four-week intervals to perform the immune simulation. The rest of the parameters were kept as default [56].

### Codon adaptation and in silico cloning

For achieving successive expression of the vaccine model in the prokaryotic (*E. coli*) system, the amino acid chain was reverse translated and optimized by applying JCAT (Java Codon Adaptation Tool) server [57]. To assess the level of expressivity in the *E. coli* system, the server calculated GC contents and Codon Adaptation Index (CAI). Three default parameters were calculated, such as a) Rho-independent termination of transcription, b) prokaryotic ribosome binding sites, and c) cleavage site for restriction enzymes [58]. Finally, we performed *in silico* cloning of the vaccine gene using the plasmid vector pET-28b(+) sequence, retrieved from the Addgene vector database. Further, we added two enzymes, BamHI and XbaI restriction sites, at both ends of the vaccine gene [59].

**Figure 5.**
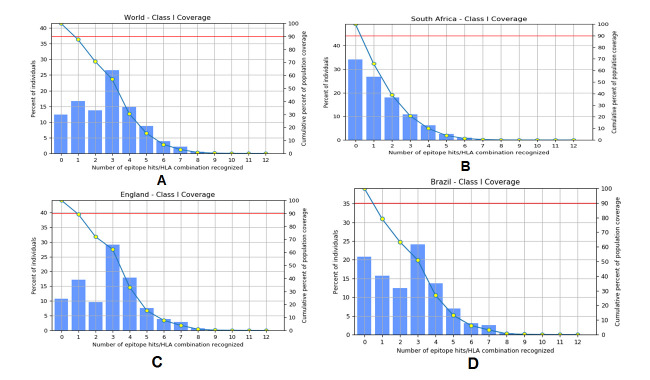
Population coverage by selected CTL epitopes and their respective MHC-I binding alleles. A) population coverage of CTL epitopes around the World, B) population coverage of CTL epitopes in South Africa, C) population coverage of CTL epitopes in England, and d) population coverage of CTL epitopes in Brazil.

## RESULTS

### Prediction and identification of CTL and HTL epitopes

The epitopic region of an antigen is a part of the immune system stimulator. Other than antibodies, B cells, or T cells, more specifically, common epitopic vaccine construct may achieve a dual purpose of boosting the host immune system. From this perspective, we selected a total of 17 CTL epitopes that have potent antigenicity. Out of 17 CTL epitopes, 5 epitopes are present in all four variants. Among 17 CTL epitopes, 2 unique CTL epitopes are present in B.1.351 lineage, and 5 unique CTL epitopes are present in the B.1.1.7 lineage. The 4 CTL epitopes, such as SQCVNLTTR, NIADYNYKL, FQPTYGVGY, SPGSASSVA are common epitopes that are present in the B.1.1.28 lineage and other variants (Table 2). However, the SQCVNLTTR epitope is not present in the B.1.351 lineage due to mutation of L>F. However, this epitope is present in the other three variants. Two 9-mer epitopes (NIADYNYKL and SPGSASSVA) are present only in B.1.351 and B.1.1.28 lineage but are absent in the other two variants. Another 9mer epitope (FQPTYGVGY) was identified from the other three newly emerging lineage, but the epitope is not present in the Wuhan variant (Table 3). Nevertheless, all these identified epitopes are not always common for all variants. The same techniques were employed for the identification and characterization of the common and variants HTL epitopes. Finally, we identified a significant 6 HTL (15-mer epitopes) from all variants. A total of 23 epitopes (17 CTL and 6 HTL) were considered for the final vaccine construct. The VaxiJen score of the CTL epitopes was calculated and is tabulated in Table 2.

**Table 2:** Common B cell derived CTL epitopes with the encountered MHC-I alleles.

Wuhan strain	B.1.351 strain	B.1.1.7 strain	B.1.1.28 strain	Alleles	Vaxijen Score
VVFLHVTYV	VVFLHVTYV	VVFLHVTYV	VVFLHVTYV	HLA-A*02:01 HLA-A*02:02 HLA-A*02:03 HLA-A*02:06 HLA-A*68:02	1.5122 (Probable ANTIGEN)
GKQGNFKNL	GKQGNFKNL	GKQGNFKNL	GKQGNFKNL	HLA-A*36:01	1.0607 (Probable ANTIGEN)
GIYQTSNFR	GIYQTSNFR	GIYQTSNFR	GIYQTSNFR	HLA-A*11:01 HLA-A*31:01 HLA-A*33:03 HLA-B*27:05	0.5380 (Probable ANTIGEN)
VSPTKLNDL	VSPTKLNDL	VSPTKLNDL	VSPTKLNDL	HLA-B*58:01 HLA-B*51	1.4610 (Probable ANTIGEN)
FKNHTSPDV	FKNHTSPDV	FKNHTSPDV	FKNHTSPDV	HLA-B*53:01 HLA-B*54:01	0.4846 (Probable ANTIGEN)
KIADYNYKL	-	KIADYNYKL	-	HLA-A*02:01 HLA-A*02:05 HLA-A*24 HLA-A*31:01 HLA-B*27:05 HLA-B*39:02	1.6639 (Probable ANTIGEN)
SQCVNLTTR	-	SQCVNLTTR	SQCVNLTTR	HLA-A*31:01 HLA-A*33:03 HLA-B*27:05	1.5476 (Probable ANTIGN)
-	-	EILDITPCS	-	HLA-A*25:01	1.0536 (Probable ANTIGEN)
-	SQCVNFTTR	-	-	HLA-A*31:01 HLA-A*33:03 HLA-B*58:01	1.7440 (Probable ANTIGEN)
-	LLALHISYL	-	-	HLA-A*02:01 HLA-A*02:05 HLA-A*24 HLA-A*03:01	1.3114 (Probable ANTIGEN)
-	NIADYNYKL	-	NIADYNYKL	HLA-A*02:01 HLA-A*02:05 HLA-A*24 HLA-A*03:01 HLA-B*27:02 HLA-B*35:01	1.5485 (Probable ANTIGEN)
-	FQPTYGVGY	FQPTYGVGY	FQPTYGVGY	HLA-B*27:02 HLA-B*27:05 HLA-B*35:01 HLA-A*43:01	0.4935 (Probable ANTIGEN)
-	SPGSASSVA	-	SPGSASSVA	HLA-B*51:01 HLA-B*52:01 HLA-B*53:01 HLA-B*54:01 HLA-B*51:01 HLA-B*51:02	0.4280 (Probable ANTIGEN)
-	-	EGFNCYFPL	-	HLA-A*02 HLA-B*14:01 HLA-B*53:01 HLA-B*51:02 HLA-B*51:01 HLA-B*40:01	0.5453 (Probable ANTIGEN)
-	-	RDIDDTTDA	-	HLA-B*40:01 HLA-B*37:01 HLA-B*44:03 HLA-B*67:01	0.7902 (Probable ANTIGEN)
-	-	FTISVTTEI	-	HLA-B*37:01 HLA-B*58:01 HLA-B*51:01 HLA-B*54:01 HLA-B*53:01 HLA-B*51:01 HLA-B*51:02	0.8535 (Probable ANTIGEN)
-	-	IITTHNTFV	-	HLA-A*02:01 HLA-A*02:05 HLA-B*53:01 HLA-B*51:01	0.4551 (Probable ANTIGEN)

### Cluster analysis of the MHC alleles

The cluster analysis of the possible HLA allele (MHC class-I and class-II) shows the interaction with their corresponding predicted epitopes, indicating a robust allelic interaction with both CTL and HTL alleles of the vaccine construct. The heatmap was predicted with a generalized format. It summarized the interaction of both alleles, which were calculated by the online tool MHCcluster 2.0. We found both strong and weaker interactions (Fig. 4A, B).

**Figure 6.**
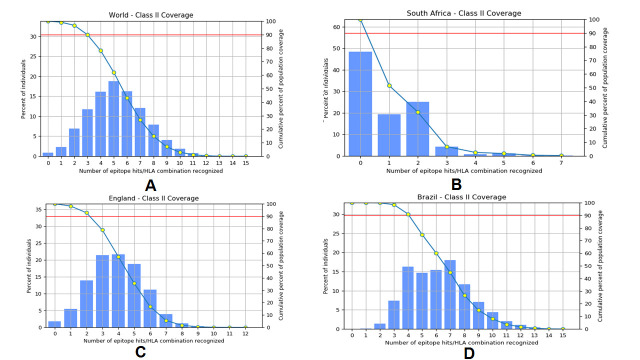
Population coverage by selected HTL epitopes and their respective MHC-II binding alleles. A) population coverage of CTL epitopes around the World, B) population coverage of CTL epitopes in South Africa, C) population coverage of CTL epitopes in England, and d) population coverage of CTL epitopes in Brazil.

### Population coverage analysis

IEDB was used for the identification of epitope-based population coverage analysis of the corresponding MHC-I and MHC-II alleles. From the server, a sharp idea of population coverage was retrieved for MHC-I and MHC-II epitopes. We have listed the coverage value of different world regions, mainly where the second wave of COVID-19 is progressing. We have also shown the population coverage concerning filtrated epitopes and responsible three emerging variants and Wuhan variant for both MHC-I and MHC-II alleles, respectively (Fig. 5 and Fig. 6).

### Allergenicity, primary and secondary structure analysis of the vaccine construct

AllerTOP 2.0 and AllergenFP v.1.0 servers were used to calculate the allergenicity of our vaccine to be safe for further use. Subsequently, servers calculated the allergenicity of C1 and compared it with our designed vaccine candidate to support the designed vaccine safety and non-allergenic characteristic.

The server predicted the physicochemical properties of the vaccine (Supplementary Table 1). The vaccine construct was found to be basic (8.98) in nature. It was observed that the theoretical Isoelectric point of the vaccine was 8.98, and it possesses a molecular weight (MW) of 52.306 KDa. GRAVY was estimated to be -0.168, the negative GRAVY value indicating the vaccine protein might be hydrophilic in nature. The hydrophilic nature of the protein should assist in the easy purification and formulation of the vaccine. Thermo-stability (Aliphatic index) of the vaccine was found to be 76.18. In addition, the half-life was calculated >30 hours, >20 hours, and >10 hours in mammalian reticulocytes (*in vitro*), yeast (*in vivo*), and *E. coli* (*in vivo*), respectively. The instability index (II) was computed and was noted as 23.36. It indicates a stable vaccine construct. The final vaccine candidate was compared with the positive control (C1), and a comparison of the physicochemical properties is recorded in Table 4. It appeared that the primary sequence of vaccine candidates has a better result in comparison with the positive control. The final vaccine construct showed suitable physicochemical properties like hydrophilicity, stability, and increased immunogenicity. At the same time, the decreased probable side effects informed us that the vaccine candidate is safer for further administration.

**Figure 7.**
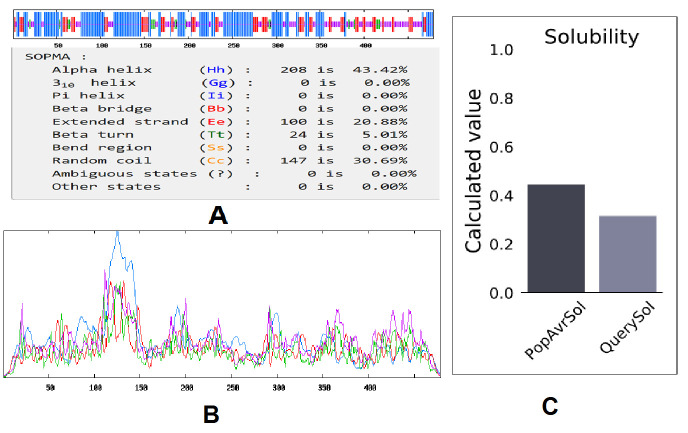
Secondary structure prediction plot of the vaccine construct. Alpha helices are shown in blue color, while extended strands and beta turns are shown by red and green colors, respectively. (A) The visualization of the prediction and (B) The score curves for each predicted state. C) Protein-Sol predicted water solubility test of the vaccine construct.

SOPMA analysis predicted the secondary structure values like the alpha helix, extended strand, beta-turn, and random coil as 43.42%, 20.88%, 5.01%, and 30.69%, respectively (Fig. 7A, B). The server was calculated with a window width value of 17. We have fixed the similarity threshold value to 8. In this case, we considered four numbers of states. Moreover, the Protein-Sol server calculated the solubility of the vaccine construct, which was found to be soluble (0.316) in the basic of scaled solubility value (QuerySol) (Fig. 7C).

**Figure 8.**
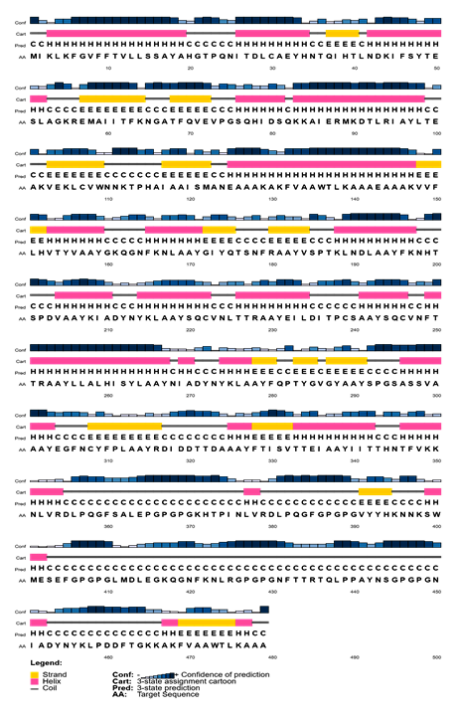
Secondary structure analysis of vaccine protein using PESIPRED server.

The quality of the secondary structure of the vaccine construct predicted through PSIPRED 4.0 server indicates a superior quality of the secondary structure of the vaccine (Fig. 8).

### Prediction of antigenicity

The analysis of antigenicity using VaxiJen v2.0 showed that the vaccine construct is a potent antigenic with an antigenicity score of 0.667. Comparative analysis of the antigenicity score of positive control (C1) and the candidate vaccine is illustrated in table 4. We have noted that the antigenicity of considered positive control is lesser than our designed vaccine. Our multi-epitopic vaccine construct with an adjuvant and PADRE sequence may stimulate a more robust immune response.

### Validation of protein structure

**Figure 9.**
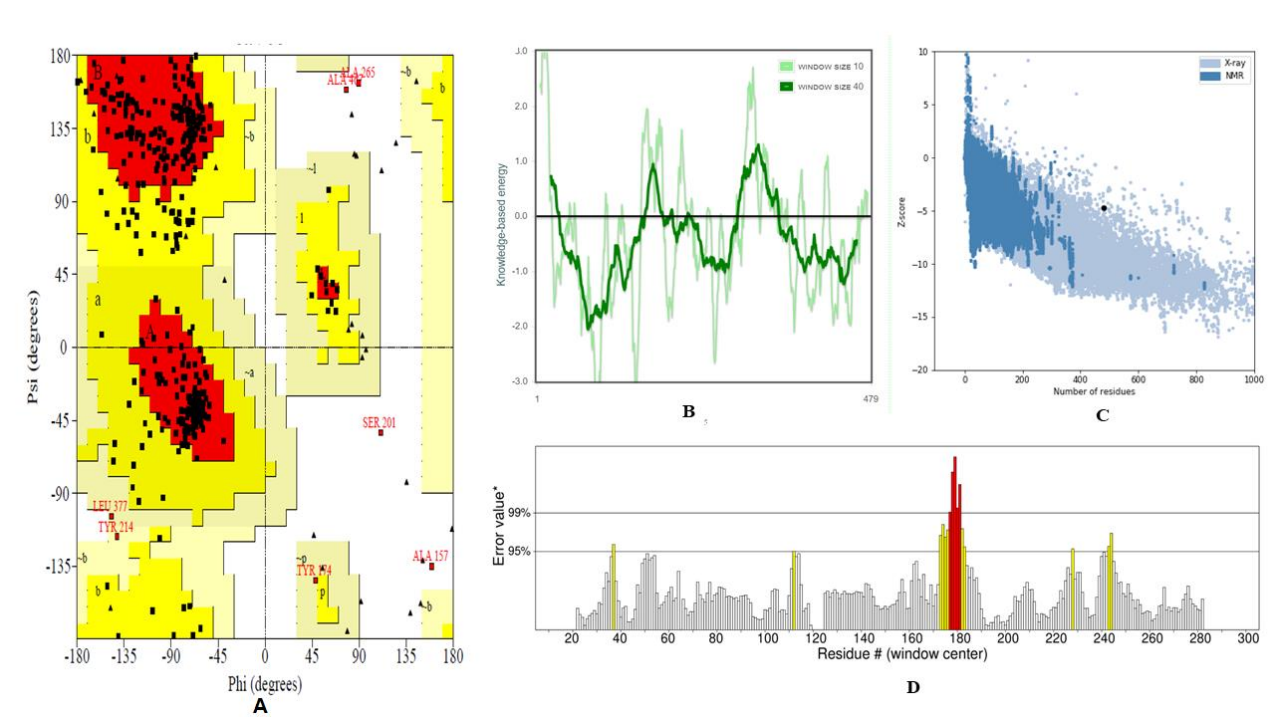
3D structure validation of vaccine construct. A) Ramachandran plot for all residues of vaccine construct. B) Energy plot of amino acid of vaccine construct element. C) The 'Z' score of vaccine construct (black dot) within the 'Z' score range of experimentally proved structure. D) ERRAT plot.

Validation of protein structure was performed using ProSA, ERRAT, and PROCHECK webserver. This analysis was to evaluate the overall quality of the protein structure of the vaccine candidate. The Ramachandran plot of the refined vaccine model showed that 83.1%, 15.3%, 0.7%, and 1.0% of the residues were present in four specific regions such as favoured, additional allowed, generously allowed, and disallowed, respectively (Fig. 9A, Table 5). The ERRAT predicted the overall quality factor to be 66.75 (Fig. 9D). The Z-score of the refined model was determined and observed as -4.75 (Fig. 9C). The negative score indicates that it is an excellent 3D protein model. We have generated another local quality model using ProSA, which is depicted through a significant plot. It showed the overall quality of the model to be good (Fig. 9B).

### Disulfide engineering of the 3D multi-epitope vaccine construct

**Figure 10.**
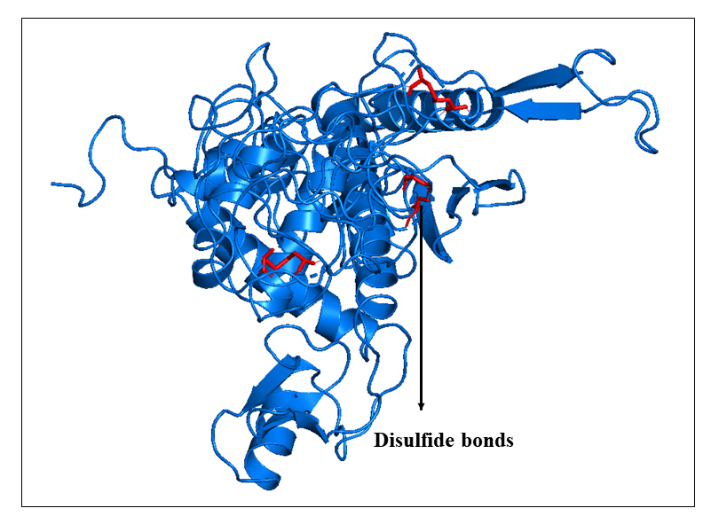
Disulfide engineering of the vaccine structure. The red bonds are the disulfide of the mutated residues to increase vaccine binding stability.

A total of 42 pairs of residues were identified to establish a disulfide bond using the DbD2 server. However, chi^3^ and B-factor energy (Kcal/mol) are the two main criteria to screen out the following residues that satisfy the bond formation, which was mutated to cysteine (C) residue (Fig. 10). Screening of the mutated residues was performed on the basis of <2.5 energy value, and the range of the chi^3^ value was set as -87 to +97 [60].

### Codon adaptation and in silico cloning

**Figure 11.**
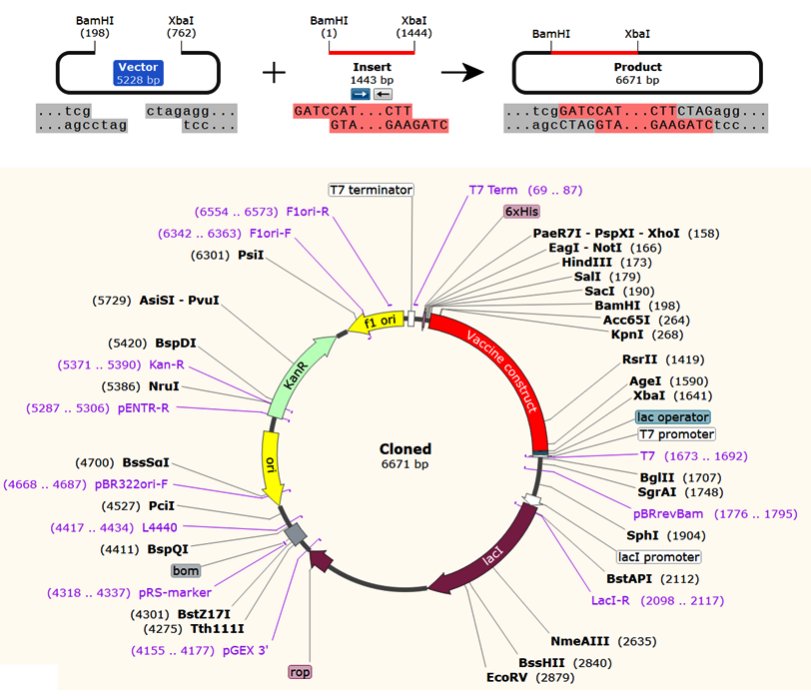
*In silico* cloning of the vaccine construct performed in *E. coli* (k12 strain, prokaryotic host) using vector pET28b (+) expression vector.

The reverse translation of the protein was transformed into the nucleotide sequence, and the codon adaptation was performed to improve cloning and expression in the *E. coli* K12 strain. The JCat server estimates the CAI-value (0.9320) and GC% (50.73%) of the optimized sequences. The cutting sites of XbaI (762) and BamHI (198) restriction enzymes were embedded for the final *in silico* cloning process at the initial and the end portion of the sequence. Finally, processed DNA sequences were inserted within the Plasmid pET28a (+) vector and are shown in Figure 11. The *in silico* cloning was performed with the help of SnapGene V. 5.2 (www.snapgene.com) [61]. The estimated total length of the final clone was 6.671 Kbp.

### Molecular docking of vaccine construct with TLR4/MD2 complex

**Figure 12.**
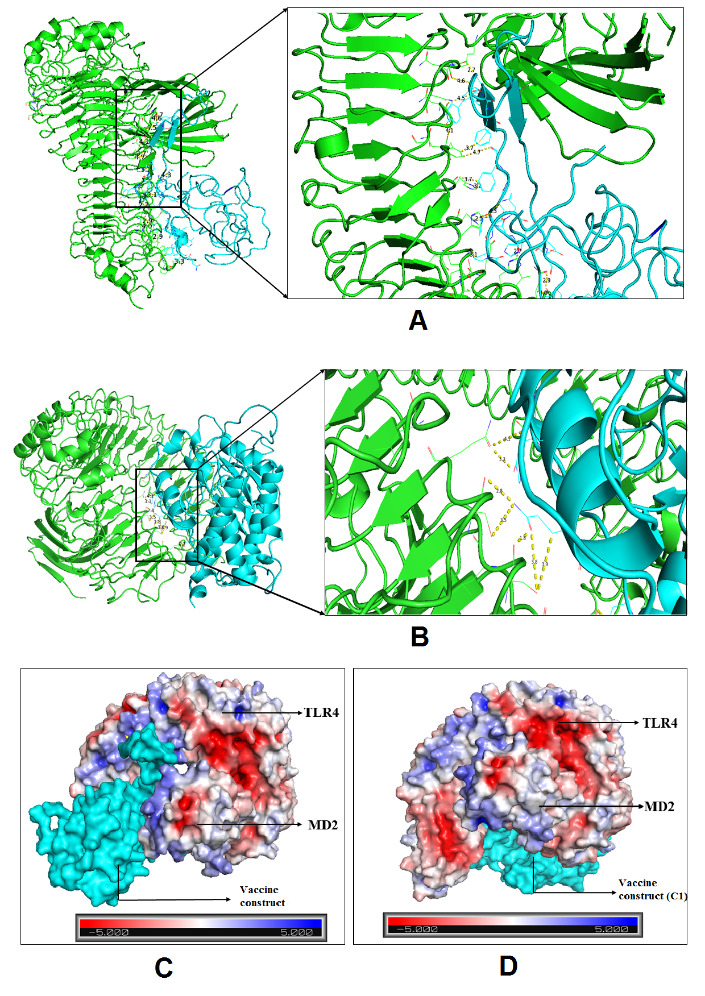
Molecular docking of peptide vaccine with TLR4/MD2 complex. A) Interaction of the vaccine candidate with TLR4/MD monomeric complex. B) Interaction of the C1 control with TLR4/MD monomeric complex. C) Docked complex of TLR4/MD2 and vaccine construct in specific receptor sites (surface structure build by APBS electrostatic energy). D) Docked complex C1 in specific receptor sites (surface structure build by APBS electrostatic energy).

Docking was performed by HDOCK online server, and human TLR4/MD2 (PDB code: 3FXI) complex was used as an innate receptor molecule that activates proinflammatory cascades. Among the top 10 models, we picked the top rank docking cluster with the highest binding score (-312.89), selected for illustration and visualization of the binding interaction (Fig. 12A). The evaluated result of comparative molecular interaction analysis of positive control (C1) against TLR4/MD2 complex is shown in Figure 12B. The generated HDOCK score between TLR4/MD2 and C1 control was -282.742. We found that the binding affinity of our designed vaccine candidate with TLR4/MD2 complex is quite stable from both the analysis. The molecular binding of the designed vaccine construct and C1 with TLR4/MD2 complex (calculated hydrogen bonds distance) is presented in Supplementary Table 2 and Supplementary Table 3.

Next, the HawkDock server provided free binding energy using MM/GBSA based machine learning approach. The free binding energy associated with the complex was -113.47 kcal/mol. Also, the free binding energy of the control was recorded as -103.23 Kcal/mol. Thus, it appears that both have excellent binding affinity with the receptor molecule, TLR4/MD2. An advanced Poisson-Boltzmann Solver (APBS) electrostatics calculation of the selected docking complex of our vaccine candidate and control is represented in Figure 12C and 12D, respectively, where the red color indicates a strong affinity, whereas the white color depicts weak interactions.

**Table 3:** Common B cell derived HTL epitopes with the encountered MHC-II alleles.

Wuhan variant		B.1.351 variant		B.1.1.7 variant		B.1.1.28 variant	
Epitopes	Alleles	Epitopes Alleles		Epitopes	Alleles	Epitopes	Alleles
NLVRDLPQGFSALEP	HLA-DRB1*01:01 HLA-DRB1*03:01 HLA-DRB1*07:01 HLA-DRB1*08:02 HLA-DRB1*09:01 HLA-DRB1*11:01 HLA-DRB1*12:01 HLA-DRB1*13:02 HLA-DRB1*15:01 HLA-DRB3*01:01 HLA-DRB3*02:02 HLA-DRB4*01:01 HLA-DRB5*01:01	NIL	NIL	NLVRDLPQGFSALEP	HLA-DRB1*01:01 HLA-DRB1*03:01 HLA-DRB1*07:01 HLA-DRB1*08:02 HLA-DRB1*09:01 HLA-DRB1*11:01 HLA-DRB1*12:01 HLA-DRB1*13:02 HLA-DRB1*15:01 HLA-DRB3*01:01 HLA-DRB3*02:02 HLA-DRB4*01:01 HLA-DRB5*01:01	NLVRDLPQGFSALEP	HLA-DRB1*01:01 HLA-DRB1*03:01 HLA-DRB1*07:01 HLA-DRB1*08:02 HLA-DRB1*09:01 HLA-DRB1*11:01 HLA-DRB1*12:01 HLA-DRB1*13:02 HLA-DRB1*15:01 HLA-DRB3*01:01 HLA-DRB3*02:02 HLA-DRB4*01:01 HLA-DRB5*01:01
KHTPINLVRDLPQGF	HLA-DRB1*04:05 HLA-DRB1*07:01 HLA-DRB3*01:01 HLA-DRB3*02:02	NIL	NIL	KHTPINLVRDLPQGF	HLA-DRB1*04:05 HLA-DRB1*07:01 HLA-DRB3*01:01 HLA-DRB3*02:02	KHTPINLVRDLPQGF	HLA-DRB1*04:05 HLA-DRB1*07:01 HLA-DRB3*01:01 HLA-DRB3*02:02
NIL	NIL	NFTTRTQLPPAYTNS	HLA-DPA1*01:03 HLA-DPB1*02:01 HLA-DPA1*03:01 HLA-DPB1*04:02 HLA-DRB1*01:01 HLA-DRB1*09:01 HLA-DRB1*08:02	NIL	NIL	NIL	NIL
NIL	NIL	NIADYNYKLPDDFTG	HLA-DRB1*09:01 HLA-DRB5*01:01 HLA-DRB1*04:05 HLA-DPA1*01:03 HLA- DPB1*02:01 HLA-DRB3*01:01 HLA-DRB1*04:05	NIL	NIL	NIADYNYKLPDDFTG	HLA-DRB1*09:01 HLA-DRB5*01:01 HLA-DRB1*04:05 HLA-DPA1*01:03 HLA- DPB1*02:01 HLA-DRB3*01:01 HLA-DRB1*04:05
VYYHKNNKSWMESEF	HLA-DRB3*02:02 HLA-DRB1*04:01 HLA-DRB1*11:01 HLA-DRB1*13:02 HLA-DRB1*09:01 HLA-DRB3*01:01 HLA-DQA1*03:01 HLA-DQA1*03:02	VYYHKNNKSWMESEF	HLA-DRB3*02:02 HLA-DRB1*04:01 HLA-DRB1*11:01 HLA-DRB1*13:02 HLA-DRB1*09:01 HLA-DRB3*01:01 HLA-DQA1*03:01 HLA-DQA1*03:02	NIL	NIL	VYYHKNNKSWMESEF	HLA-DRB3*02:02 HLA-DRB1*04:01 HLA-DRB1*11:01 HLA-DRB1*13:02 HLA-DRB1*09:01 HLA-DRB3*01:01 HLA-DQA1*03:01 HLA-DQA1*03:02
LMDLEGKQGNFKNLR	HLA-DRB1*01:01 HLA-DRB1*04:05 HLA-DRB1*07:01 HLA-DRB1*09:01 HLA-DRB1*015:01 HLA-DRB5*01:01 HLA-DRB1*03:01 HLA-DRB1*04:01 HLA-DRB1*08:02 HLA-DRB1*11:01 HLA-DRB1*12:01 HLA-DRB1*13:02 HLA-DRB3*01:01 HLA-DRB3*02:02 HLA-DRB4*01:01	LMDLEGKQGNFKNLR	HLA-DRB1*01:01 HLA-DRB1*04:05 HLA-DRB1*07:01 HLA-DRB1*09:01 HLA-DRB1*015:01 HLA-DRB5*01:01 HLA-DRB1*03:01 HLA-DRB1*04:01 HLA-DRB1*08:02 HLA-DRB1*11:01 HLA-DRB1*12:01 HLA-DRB1*13:02 HLA-DRB3*01:01 HLA-DRB3*02:02 HLA-DRB4*01:01	LMDLEGKQGNFKNLR	HLA-DRB1*01:01 HLA-DRB1*04:05 HLA-DRB1*07:01 HLA-DRB1*09:01 HLA-DRB1*015:01 HLA-DRB5*01:01 HLA-DRB1*03:01 HLA-DRB1*04:01 HLA-DRB1*08:02 HLA-DRB1*11:01 HLA-DRB1*12:01 HLA-DRB1*13:02 HLA-DRB3*01:01 HLA-DRB3*02:02 HLA-DRB4*01:01	LMDLEGKQGNFKNLR	HLA-DRB1*01:01 HLA-DRB1*04:05 HLA-DRB1*07:01 HLA-DRB1*09:01 HLA-DRB1*015:01 HLA-DRB5*01:01 HLA-DRB1*03:01 HLA-DRB1*04:01 HLA-DRB1*08:02 HLA-DRB1*11:01 HLA-DRB1*12:01 HLA-DRB1*13:02 HLA-DRB3*01:01 HLA-DRB3*02:02 HLA-DRB4*01:01

### Molecular dynamics simulation

**Figure 13.**
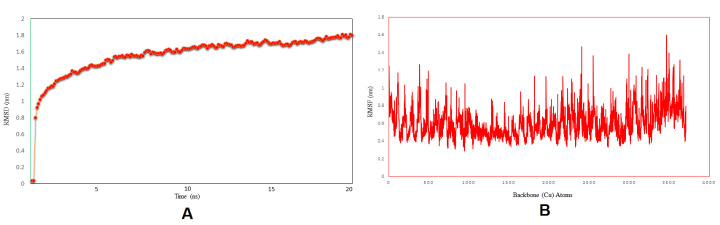
MD simulation of the vaccine TLR4/MD2 complex. (A) The RMSD plot of TLR4/MD2 and vaccine construct (B) In the RMSF plot, the peaks represent regions with a high degree of flexibility.

The TLR4/MD2-vaccine complex stabilized about 8 ns in the 20 ns long MD simulation (Fig. 13A). The Root Mean Square Deviation (RMSD) was calculated to determine the structural stability of the TLR4/MD2-vaccine complex. Additionally, RMSF was experiential value to evaluate the side-chain flexibility. The RMSF value ranged from 0.3 nm to 1.6 nm, and the maximum peaks at ~1.6 nm in the plot indicate highly flexible regions in the complex, as represented in Figure 13B.

### Normal mode analysis

**Figure 14.**
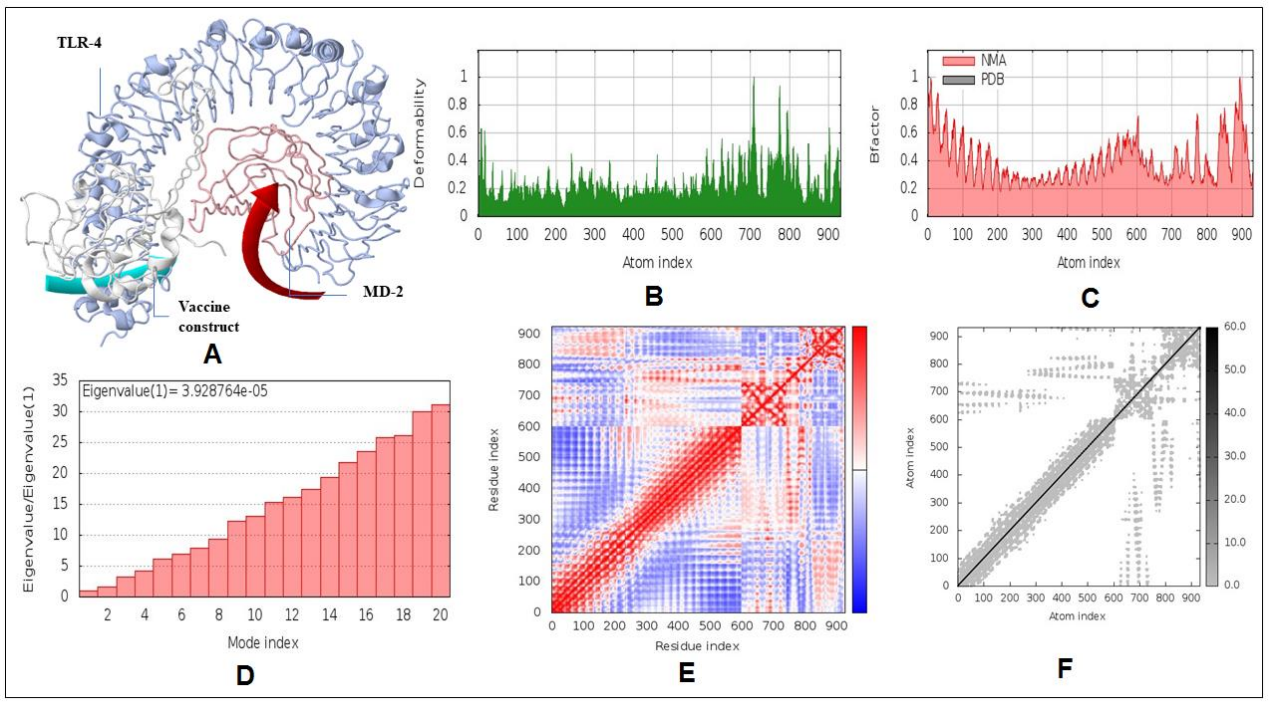
NMA analysis of docked complex. A) Mobility of the docking complex vaccine construct and TLR4/MD2 indicated with arrows. B) Deformability plot of the docking complex. C) Calculated B-factor of NMA and PDB B-factor. D) Eigenvalue for the docking complex, E) Covariance matrix map of atomic pair of residues. F) Connection spring map of the elastic network model.

Figure 14A demonstrates the normal mode analysis of TLR4/MD2 and vaccine candidate complex. The peaks in the deformability graph show the corresponding regions in the protein with deformability (Fig. 14B). The B-factor plot of the complex shows a definite visualization and demonstrates the assessment between the NMA and the PDB (Fig. 14C). The eigenvalue of the complex is 3.928764e-^05^ and is showed in Figure 14D. The covariance map of the complex shows the interacting motion between the two molecules. Here the correlated motion between a pair of residues is indicated by red color, uncorrelated motion is indicated by white color, and anti-correlated motion is indicated by blue color (Fig. 14E). The elastic map of the complex represents the connection between the atoms of the large molecules, and darker grey regions indicate stiffer regions (Fig. 14F).

**Table 4:** Comparative analysis of the physicochemical, allergenicity, and antigenicity properties of the positive vaccine control (C1) and designed SARS-CoV-2 vaccine candidate.

Properties	Parameters	Value	
		C1	Vaccine candidate
Physicochemical	Molecular weight	51.64 KDa	52.30 KDa
	Instability index (II)	27.09	23.36
	Aliphatic index	79	76.18
	Isoelectric point (pI)	10	8.98
	GRAVY	-0.354	-0.168
Allergenicity	AllerTOP V2.0 and AllergenFP v.1.0	Non-allergenic	Non-allergenic
Antigenicity	VaxiJen 2.0	0.59	0.6679

### Immune simulation profiling

**Figure 15.**
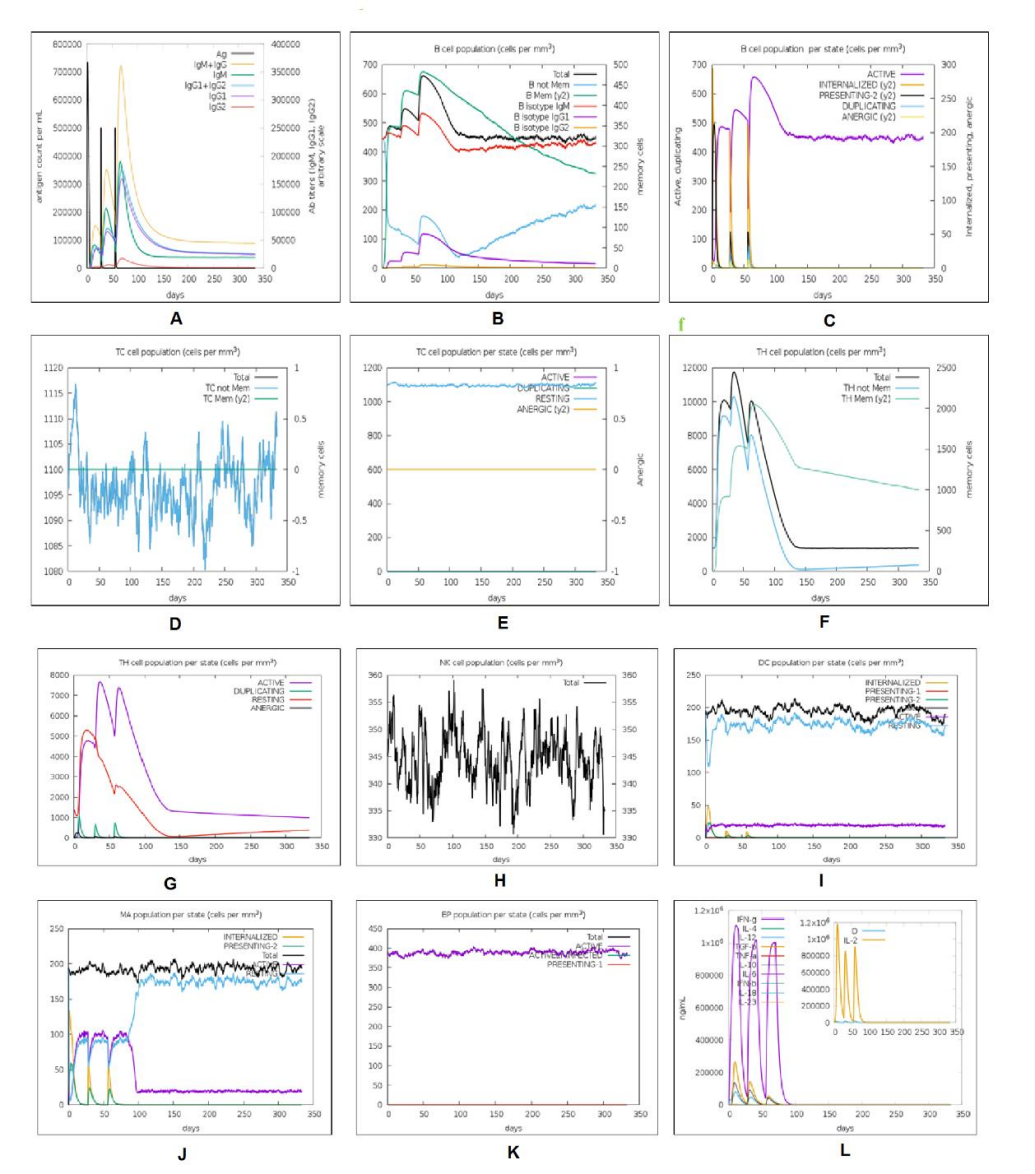
*In silico* immune response simulation of the positive control (C1) through C-ImmSim program. A) Elevation of immunoglobulins at different concentration of antigen, B) Population of B lymphocytes (IgM, IgG1 and IgG2) after three injections, C) population of B cell per entity-state (i.e., showing counts for active, presenting on class-II, internalized the Ag, duplicating and anergic by the different color variant. D) Amount of Cytotoxic T lymphocytes population, E) Cytotoxic T lymphocytes population in different states; resting and active. F) Total count of TH cell population along with memory cells, and sub-divided in isotypes IgM, IgG1, and IgG2. G) Population per entity-state of Helper T-cell count in the resting and active states. H) Population of Natural Killer cells. I) Population of Dendritic cells in the active and resting states. J) Population of Macrophages. K) Total count of EC cells that are broken down to active, virus-infected, and presenting on class-I MHC molecule. L) Concentration of cytokines and interleukins with Simpson index.

**Figure 16.**
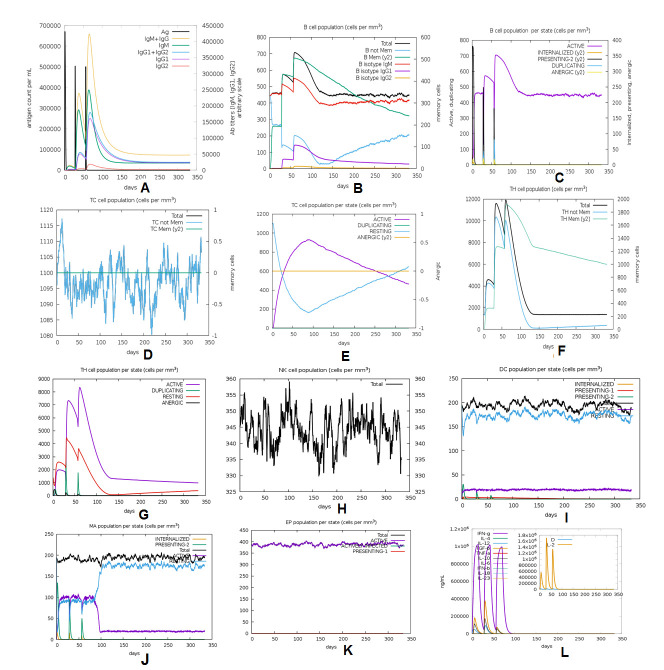
*In silico* immune response simulation of the new proposed novel vaccine construct. A) Elevation of immunoglobulins at different concentration of antigen, B) Population of B lymphocytes (IgM, IgG1 and IgG2) after three injections, C) population of B lymphocytes per entity-state (i.e., showing counts for active, presenting on class-II, internalized the Ag, duplicating and antigenic by the different color variant. D) Amount of Cytotoxic T lymphocytes population, E) Cytotoxic T lymphocytes population in different states; resting and active. F) Total count of TH cell population along with memory cells, and sub-divided in isotypes IgM, IgG1, and IgG2. G) Population per entity-state of Helper T cell count in the resting and active states. H) Population of Natural Killer cells. I) Population of Dendritic cells in the active and resting states. J) Population of Macrophages. K) Total count of EC cells that is broken down to active, virus-infected, and presenting on class-I MHC molecule. L) Concentration of cytokines and interleukins with Simpson index.

The *in silico* immune simulation of the vaccine construct was performed with primary and secondary immune responses by triggering the immune system, including CTL, HTL, sustainable memory cells, and other immune cells (NK cells, DC cells etc.). The levels of IgM+IgG immunoglobulin were radically rising after the administration of the vaccine candidate, and prolonged immune responses were observed against SARS-CoV-2 by the high titers of IgG and IgM immunoglobulin (primary immune responses). Furthermore, the C1 positive control was used for comparative assessment of the vaccine candidate immune simulation (Fig. 15A). A high resemblance was shown between the immune simulation results of the C1 and our next-generation vaccine construct (Fig. 15 and Fig. 16). The innate immunity of the host body (after administration of vaccine) along with B cell population in both acting and resting phase was found to be increased with B isotype IgM and B-memory cell. It might scale up to 600-700 cells/mm^3^ (Fig. 16B and C). The control shows a similar type of increment of B isotype IgM and B-memory cells (Fig. 15B and C). Moreover, the elevation of CTL cells reached a maximum of 1117 cells/mm^3^ after ~8 days of vaccine immunization and gradually decreased after 25 days (Fig. 16D). Additionally, at the state of the active and resting phase, the T cell populations showed great diversity in their cell population (Fig. 16E). C1 shows a similar level of CTL cell elevation both in the active and resting phase, represented in Figure 15d-15e. In response to both control and the vaccine, elevated HTL cells (both active and resting phases) play a key role in the establishment of adaptive immunity against virus infections. Subsequently, the elevated HTL cells generated a high number of memory cells (Fig. 15F, G and 16F and G). Memory cells play a vital role in regulating and preventing viral infection/reinfection through self-memorization upon encountering pathogens. The administration of both C1 and designed vaccine successfully elevated other central regulators of the immune system (cytokines, interleukins, and NK cells) (Fig. 15H-15L and 16H-16L). This comparative study signifies the designed vaccine as a potent next-generation vaccine for eliciting a robust immune response to SARS-CoV-2 infection.

**Table 5:** Distribution of amino acid residues showing in Ramachandran plot.

Amino acid position	Residue number	Percentage (%)	Total
Favoured region	348	83.1%	419
Addition allowed region	64	15.3%	
Generously allowed region	3	0.7%	
Disallowed region	4	1.0%	
End-residues			2
Glycine residues			32
Proline residues			26
Total residues			479

## DISCUSSION

The ongoing COVID-19 surge has become a global crisis and urgently requires a next-generation effective vaccine to protect against all the significant viruses [62,63]. Nowadays, the COVID-19 situation is more devastating in various developed and developing countries such as India, Italy, Germany, UK, the USA, Brazil etc. [64]. Emerging new variants of SARS-CoV-2 are affecting the populations, and the infection rate is sharply increasing [65]. The significant mutant variants such as B.1.1.7, B.1.351, B.1.1.28 lineage of SARS-CoV-2 are now enlisted as VOC (Variant of Concern) by WHO [66]. As the structure-function is the impact of the nonspecific mutations, the emergence of these three new variants is of urgent concern; it would likely impact key functionalities associated with COVID-19 infection, including disease severity, transmissibility, and diagnostic sensitivity along with specificity and vaccine-induced protection. Although some vaccines have already been approved for the COVID-19 Wuhan variant, no vaccine is available to protect the mutant variants and the Wuhan variant. Therefore, there is an urgent need for a next-generation vaccine to provide comprehensive protection against all these variants.

This study designed a next-generation multi-epitope peptide vaccine targeting the S-protein of SARS-CoV-2, covering all the three significant variants’ epitopes along with the Wuhan variant epitopes. Our vaccine construct has considered all significant mutations such K417N, T716I, S982A, N501Y, D118H, A570D, L18F, D614G, T1027I, P681H, H655Y, E484K, D215G, L18F, and K41N. For generating a multi-epitope peptide vaccine, B cell-derived T cell epitopes were identified, which can provide a robust immune response against SARS-CoV-2. Our vaccine construct comprises 17 (9mer) CTL epitopes and 6 HTL epitopes (15mer) and is 479 amino acids long due to the addition of adjuvant CTxB, PADRE sequence, and a peptide linker. The adjuvant was also joined with the EAAAK linker at the N-terminal of the vaccine construct, which was merged for active separation of a bifunctional fusion protein [67]. The EAAAK linker was joined with the PADRE sequence. The PADRE sequence act as a helper T cell epitope which is significant for enhancing CTL responses of different antigens. Moreover, GPGPG and KK linkers were fused with CTL and HTL epitopes, respectively.

According to the web servers, the final vaccine construct was basic and stable in the physiological pH range. Additionally, the aliphatic index score indicated that our vaccine candidate is more thermostable, the outcome of the comparative study of positive control (C1) and our vaccine construct. [68]. At the same time, the negative value of GRAVY specified its hydrophilic character, suggesting strong interactions with water molecules [69]. Therefore, the vaccine construct is water-soluble [70].

In this study, the 3D structure of the vaccine candidate was developed through the Raptor-X web server, and it was then validated through ProSA, PROCHECK, and ERRAT error plot webserver. Subsequently, ‘Z’ score and local energy plot were evaluated to comprehend its proper folding. Ramachandran plot of each amino acid residues of vaccine candidate was studied. ProSA predicted ‘Z’ score negative value (-4.75), indicating a good sign for model quality assessment [52, 71]. The local quality model analysis also indicates it as a reliable structure.

Our further analysis revealed that the vaccine construct has a strong binding affinity with TLRs. The TLRs play a substantial role in identifying pathogens and stimulating the innate immune response against pathogens [72-75]. In this study, we performed a comparative molecular docking study using the TLR4/MD2 complex with C1 and vaccine candidate with the help of HDOCK and HawkDock servers. At the same time, we have performed disulfide engineering and added Cysteine (C) residues at their satisfying position. The negative docking scores of both TLR4/MD2 with control and TLR4/MD2 vaccine candidates illustrated that the vaccine construct has similar binding interaction properties with the TLR4/MD2 complex. It implies that the vaccine can trigger TLR4 activation and enhance immune responses against a broad range of SARS-CoV-2 variants. The free binding energy of the vaccine and TLR4/MD2 complex is -113.47 kcal/mol, which indicates an excellent binding affinity between the immune sensing (TLR4) receptor and the vaccine model. The advanced APBS electrostatics calculation of the selected docking complex also supports the binding affinity of the vaccine construct with TLR4/MD2 complex.

Molecular dynamic experiments provide information on the stability of the vaccine and TLR4/MD2 complex. In this case, the RMSD results show the stability of the vaccine-human TLR4/MD2 complex after the 8ns of time scale (Fig. 13A). Similarly, RMSF shows the fluctuations of the binding interaction, and it supports the higher flexibility of this binding complex. The maximum peaks were observed at ~1.6 nm in the plot, indicating highly flexible regions in the complex (Fig. 10B). The NMA study was performed to analyze the molecular motion, comparative deformability, and B factor with PDB value. The eigenvalue was calculated as 3.928764e-^05^, which indicates the better flexibility of the vaccine candidate with immune receptor molecule. The immune simulation profiling of C1 is similar with the final vaccine construct; therefore, both are able to activate cellular and humoral immune responses. In this case, our next-generation vaccine construct with alternative multi-epitopes activated cellular and humoral immune responses against all broad range of variants of SARS-CoV-2. Furthermore, the secondary response created by the vaccine was significantly higher than the primary response. Finally, the SnapGene cloning software was performed to amplify the desired vaccine sequence within the pET28b (+) expression vector for large-scale production.

Our multi-epitopic peptide-based vaccine construct is highly significant in the current perspective compared to other reported vaccine constructs. We used all significant epitopes from the newly emerging variants of variant of concer (VOC) of the SARS-CoV-2 and Wuhan variant. This vaccine candidate may be helpful to generate the different types of neutralizing antibodies (nAb) for the protection against all VOC and end the pandemic.

## Conclusion

This study designed a novel next-generation multi-epitopic vaccine candidate against newly emerging variants of SARS-CoV-2 (B.1.1.7, B.1.351, B.1.1.28 and Wuhan variant) by using various immunoinformatics and bioinformatics. Our planned vaccine candidate passed all satisfactory conditions like physiochemical properties, antigenicity, and non-allergenicity. From this result, we concluded that our vaccine construct is safe and might be administrated into the human body after the successful pre-clinical trial and clinical trial. Further, to understand the structural validation of the vaccine candidate and the binding affinity on its immune receptor, we performed molecular docking analysis and MD simulation. The vaccine construct can stimulate a good immune response, as confirmed by immunoinformatics. Therefore, our developed next-generation vaccine construct can provide a wide range of protection against all the significant variants of SARS- CoV-2. However, *in vitro and in vivo* validation is required to ensure the vaccine construct efficacy through animal models and human clinical trials. This designed vaccine candidate might contribute to controlling the next COVID-19 wave and the pandemic situation on a global scale.

## Supplementary Materials

The Supplementary data can be found online at: www.aginganddisease.org/EN/10.14336/AD.2021.0517.


