# Supplementary material for: A Next-Generation Vaccine Candidate Using Alternative Epitopes to Protect against Wuhan and All Significant Mutant Variants of SARS-CoV-2: An Immunoinformatics Approach
Source: Aging Dis. 2021 Dec 1;12(8):2173–95. doi: 10.14336/AD.2021.0518 (PMC8612605; doi:10.14336/AD.2021.0518)
Supplement: Supplementary file 1 [file AD-12-8-2173-s.pdf]

SUPPLEMENTARY DATA

A Next-Generation Vaccine Candidate Using Alternative Epitopes to Protect against Wuhan and All Significant Mutant Variants of SARS-CoV-2: an Immunoinformatics Approach

Manojit Bhattacharya[1,#], Ashish Ranjan Sharma[2,#], Pratik Ghosh[3], Sang-Soo Lee[2*], Chiranjib Chakraborty [4*]

© 2021. Bhattacharya M et al. Published online at http://www.aginganddisease.org/EN/10.14336/AD.2021.0517

SUPPLEMENTARY DATA

Supplementary Table 1. Antigenicity, allergenicity, solubility, and physicochemical property assessments of the primary sequence of multi-epitope-based vaccine construct against SARS-CoV-2.

Sl. No.	Features	Assessment
1.	Antigenicity	0.6679 (Probable ANTIGEN) by VaxiJen v2.0
2.	Allergenicity	Probable non-allergen (AllerTOP v.2.0) and<br>Probable non-allergen (AllergenFP v.1.0)
3.	Solubility	0.316<br>(Soluble)
4.	Number of amino acids	479
5.	Molecular weight	52306.41 dalton
6.	Theoretical Isoelectric point (pI)	8.98
7.	Total number of atoms	7318
8.	Formula	$C_{2387}H_{3617}N_{615}O_{687}S_{12}$
9.	Estimated half-life	30 hours (mammalian reticulocytes, in vitro)<br>>20 min (yeast, *in vivo*)<br>>10 hours (*Escherichia coli*, *in vivo*)
10.	Instability index	23.36<br>(Stable)
11.	Aliphatic index	76.18
12.	Grand average of hydropathicity (GRAVY)	-0.168

© 2021. Bhattacharya M et al. Published online at http://www.aginganddisease.org/EN/10.14336/AD.2021.0517

SUPPLEMENTARY DATA

Supplementary Table 2. Molecular docking of TLR4/MD2 and vaccine construct showing the interface residues where they bind along with distance measurement.

**Receptor ligands interactions**	**Distance (A^0)**
361A - 311	4.245
362A - 311	2.662
362A 327	4.326
363A - 311	3.785
363A - 313	4.991
365A - 311	3.416
365A - 313	4.068
383A - 311	4.537
384A - 310	4.210
386A - 315	4.875
408A - 306	4.657
408A - 308	3.654
409A - 310	4.535
431A - 303	4.997
431A - 304	3.815
431A - 305	4.731
431A - 306	2.132
456A - 303	2.503
456A - 304	1.964
456A - 306	4.253
456A - 370	4.316
458A - 306	4.974
458A - 368	4.847
458A - 369	2.145
458A - 370	3.761
458A - 371	4.300
459A - 369	4.363
480A - 304	3.957
480A - 369	2.142
480A - 370	3.763
481A - 369	3.416
482A - 369	3.925
504A - 303	4.366
505A - 302	3.619
505A - 303	2.539
505A - 304	2.842
505A - 369	4.758
505A - 372	4.566
507A - 368	4.067
507A - 369	4.090

© 2021. Bhattacharya M et al. Published online at http://www.aginganddisease.org/EN/10.14336/AD.2021.0517

SUPPLEMENTARY DATA

Supplementary Table 3. Molecular docking of TLR4/MD2 and C1 control shows the interface residues where they bind along with distance measurement.

Receptor ligands interactions	Distance (A^0)
34A - 115	4.762
34A - 117	4.190
35A - 114	4.980
35A - 115	4.927
35A - 117	2.123
35A - 225	3.539
35A - 226	4.340
36A - 225	4.820
36A - 226	2.971
54A - 281	3.767
54A - 282	2.365
55A - 224	3.382
55A - 226	4.176
55A - 282	2.966
57A - 226	4.486
57A - 227	2.222
57A - 228	3.980
57A - 229	4.570
57A - 230	2.268
78A - 278	4.119
78A - 282	3.964
79A - 227	4.369
79A - 228	3.276
79A - 229	4.917
79A - 278	3.174
79A - 282	3.517
81A - 227	4.943
81A - 229	4.869
81A - 230	2.948
102A - 240	4.689
102A - 243	1.784
102A - 274	4.964
102A - 278	4.217
103A - 22	3.521
103A - 229	4.623
103A - 236	4.270
103A - 240	2.848
103A - 241	3.470
103A - 244	4.741
103A - 278	1.446
105A - 232	3.820
105A - 236	3.431
126A - 239	4.736
126A - 243	2.637
126A - 271	3.294

© 2021. Bhattacharya M et al. Published online at http://www.aginganddisease.org/EN/10.14336/AD.2021.0517

SUPPLEMENTARY DATA

Supplementary files

Table S1. The B cell epitopes of the Wuhan variant of SARS-CoV-2 S protein along with their length predicted by ABCPred server.

Rank	Sequence	Start position	Score
1	VTLADAGFIKQYGDCLGDIA	826	0.90
1	IGAEHVNNSYECDIPIGAGI	651	0.90
1	TNVYADSFVIRGDEVRQIAP	393	0.90
1	VYYHKNNKSWMESEFRVYSS	143	0.90
2	IHVSGTNGTKRFDNPVLPFN	68	0.89
2	DVNCTEVPVAIHADQLTPTW	614	0.89
2	SYLTPGDSSSGWTAGAAAYY	247	0.89
3	KHTPINLVRDLPQGFSALEP	206	0.88
3	VVIKVCEFQFCNDPFLGVYY	126	0.88
4	DRLITGRLQSLQTYVTQQLI	994	0.87
4	PFGEVFNATRFASVYAWNRK	337	0.87
4	VTQQLIRAAEIRASANLAAT	1008	0.87
5	DNTFVSGNCDVVIGIVNNTV	1118	0.86
5	TTLDSKTQSLLIVNNATNVV	108	0.86
6	YQPYRVVVLSFELLHAPATV	505	0.85
6	RSSVLHSTQDLFLPFFSNVT	44	0.85
6	KRISNCVADYSVLYNSASFS	356	0.85
6	IWLGFIAGLIAIVMVTIMLC	1216	0.85
7	NVLYENQKLIANQFNSAIGK	914	0.84
7	RGVYYPDKVFRSSVLHSTQD	34	0.84
8	ISSVLNDILSRLDKVEAEVQ	973	0.83
8	AGTITSGWTFGAGAALQIPF	879	0.83
8	ISVTTEILPVSMTKTSVDCT	720	0.83
8	PCSFGGVSVITPGTNTSNQV	589	0.83
9	QDKNTQEVFAQVKQIYKTPP	774	0.82
9	QQFGRDIADTTDAVRDPQTL	563	0.82
9	KNKCVNFNFNGLTGTGVLTE	535	0.82
9	SNLKPFERDISTEIYQAGST	459	0.82
9	DCALDPLSETKCTLKSFTVE	290	0.82
9	DLEGKQGNFKNLREFVFKNI	178	0.82
9	VSQPFLMDLEGKQGNFKNLR	171	0.82
9	YSSANNCTFEYVSQPFLMDL	160	0.82
10	PVAIHADQLTPTWRVYSTGS	621	0.81
10	GCVIAWNSNNLDSKVGGNYN	431	0.81
11	ECSNLLLQYGSFCTQLNRAL	748	0.80
11	GVSPTKLNDLCFTNVYADSF	381	0.80
12	LVKQLSSNFGAISSVLNDIL	962	0.79
12	GVYFASTEKSNIIRGWIFGT	89	0.79
12	CGKGYHLMSFPQSAPHGVVF	1043	0.79
13	ASQSIIAYTMSLGAENSVAY	688	0.78
13	NSPRRARSVASQSIIAYTMS	679	0.78
13	NIDGYFKIYSKHTPINLVRD	196	0.78
14	GDIAARDLICAQKFNGLTVL	842	0.77
14	DPSKPSKRSFIEDLLFNKVT	808	0.77
14	SVDCTMYICGDSTECSNLLL	735	0.77
15	VVNQNAQALNTLVKQLSSNF	951	0.76
15	KQYGDCLGDIAARDLICAQK	835	0.76
15	LLHAPATVCGPKKSTNLVKN	517	0.76
15	LLKYNENGTITDAVDCALDP	276	0.76
15	GLIAIVMVTIMLCCMTSCCS	1223	0.76
15	SGINASVVNIQKEIDRLNEV	1170	0.76

© 2021. Bhattacharya M et al. Published online at http://www.aginganddisease.org/EN/10.14336/AD.2021.0517

16	RQIAPGQTGKIADYNYKLPD	408	0.75
16	ITRFQTLLALHRSYLTPGDS	235	0.75
16	SQCVNLTTRTQLPPAYTNSF	13	0.75
17	ITPGTNTSNQVAVLYQDVNC	598	0.74
17	LTGTGVLTESNKKFLPFQQF	546	0.74
17	LYNSASFSTFKCYGVSPTKL	368	0.74
17	KSFTVEKGIYQTSNFRVQPT	304	0.74
17	LIVNNATNVVIKVCEFQFCN	118	0.74
17	VVIGIVNNTVYDPLQPELDS	1128	0.74
18	CTQLNRALTGIAVEQDKNTQ	760	0.73
18	YFPLQSYGFQPTNGVGYQPY	489	0.73
18	FSALEPLVDLPIGINITRFQ	220	0.73
18	KSWMESEFRVYSSANNCTFE	150	0.73
18	CSCGSCCKFDEDDSEPVLKG	1248	0.73
19	GSNVFQTRAGCLIGAEHVNN	639	0.72
19	VTIMLCCMTSCCSCLKGCCS	1230	0.72
19	FPREGVFVSNGTHWFVTQRN	1089	0.72
20	TPTWRVYSTGSNVFQTRAGC	630	0.71
20	GKIADYNYKLPDDFTGCVIA	416	0.71
20	GTITDAVDCALDPLSETKCT	283	0.71
20	KEELDKYFKNHTSPDVDLGD	1149	0.71
21	SALGKLQDVVNQNAQALNTL	943	0.70
21	QIYKTPPIKDFGGFNFSQIL	787	0.70
21	SKVGGNYNYLYRLFRKSNLK	443	0.70
21	TSCCSCLKGCCSCGSCCKFD	1238	0.70
22	QPTESIVRFPNITNLCPFGE	321	0.69
22	FKNHTSPDVDLGDISGINAS	1156	0.69
22	NTVYDPLQPELDSFKEELDK	1135	0.69
22	HWFVTQRNFYEPQIITTDNT	1101	0.69
23	SSGWTAGAAAYYVGYLQPRT	255	0.68
23	GQSKRVDFCGKGYHLMSFPQ	1035	0.68
24	SNKKFLPFQQFGRDIADTTD	555	0.66
25	PKKSTNLVKNKCVNFNFNGL	527	0.65
25	QELGKYEQYIKWPWYIWLGF	1201	0.65
26	FNSAIGKIQDSLSSTASALG	927	0.64
26	WTFGAGAALQIPFAMQMAYR	886	0.64
27	IDRLNEVAKNLNESLIDLQE	1183	0.63
28	QKFNGLTVLPPLLTDEMIAQ	853	0.62
29	IKDFGGFNFSQILPDPSKPS	794	0.61
29	VPAQEKNFTTAPAICHDGKA	1068	0.61
30	EIYQAGSTPCNGVEGFNCYF	471	0.60
30	ICHDGKAHFPREGVFVSNGT	1081	0.60
31	AYSNNSIAIPTNFTISVTTE	706	0.59
32	ECDIPIGAGICASYQTQTNS	661	0.58
32	FPQSAPHGVVFLHVTYVPAQ	1052	0.58
33	KMSECVLGQSKRVDFCGKGY	1028	0.57
34	EIRASANLAATKMSECVLGQ	1017	0.56
35	IQDSLSSTASALGKLQDVVN	934	0.54

SUPPLEMENTARY DATA

Table S2. The B cell epitopes of the newly emerging variant (B.1.135) of SARS-CoV-2 along S protein with their length predicted by ABCPred server.

Rank	Sequence	Start position	score
1	VTLADAGFIKQYGDCLGDIA	826	0.90
1	IGAEHVNNSYECDIPIGAGI	651	0.90
1	TNVYADSFVIRGDEVRQIAP	393	0.90
1	VYYHKNNKSWMESEFRVYSS	143	0.90
2	IHVSGTNGTKRFANPVLPFN	68	0.89
3	KHTPINLVRGLPQGFSALEP	206	0.88
3	VVIKVCEFQFCNDPFLGVYY	126	0.88
4	DRLITGRLQSLQTYVTQQLI	994	0.87
4	GVNCTEVPVAIHADQLTPTW	614	0.87
4	PCSFGGVSVITPGTNTSNQV	589	0.87
4	PFGEVFNATRFASVYAWNRK	337	0.87
4	VTQQLIRAAEIRASANLAAT	1008	0.87
5	SYLTPGDSSSGWTAGAAAYY	247	0.86
5	DNTFVSGNCDVVIGIVNNTV	1118	0.86
5	TTLDSKTQSLLIVNNATNVV	108	0.86
6	KQYGDCLGDIAARDLICAQK	835	0.85
6	RSSVLHSTQDLFLPFFSNVT	44	0.85
6	KRISNCVADYSVLYNSASFS	356	0.85
6	APRDGQAYVRKDGEWVLLST	1216	0.85
7	NVLYENQKLIANQFNSAIGK	914	0.84
7	RGVYYPDKVFRSSVLHSTQD	34	0.84
8	ISSVLNDILSRLDKVEAEVQ	973	0.83
8	AGTITSGWTFGAGAALQIPF	879	0.83
8	ISVTTEILPVSMTKTSVDCT	720	0.83
8	PVAIHADQLTPTWRVYSTGS	621	0.83
9	QDKNTQEVFAQVKQIYKTPP	774	0.82
9	ITPGTNTSNQVAVLYQGVNC	598	0.82
9	QQFGRDIADTTDAVRDPQTL	563	0.82
9	KNKCVNFNFNGLTGTGVLTE	535	0.82
9	SNLKPFERDISTEIYQAGST	459	0.82
9	DLEGKQGNFKNLREFVFKNI	178	0.82
9	VSQPFLMDLEGKQGNFKNLR	171	0.82
9	YSSANNCTFEYVSQPFLMDL	160	0.82
10	GVYFASTEKSNIIRGWIFGT	89	0.81
10	YQPYRVVVLSFELLHAPATV	505	0.81
10	GCVIAWNSNNLDSKVGGNYN	431	0.81
11	ECSNLLLQYGSFCTQLNRAL	748	0.80
11	GVSPTKLNDLCFTNVYADSF	381	0.80
12	LVKQLSSNFGAISSVLNDIL	962	0.79
12	CGKGYHLMSFPQSAPHGVVF	1043	0.79
13	ASQSIIAYTMSLGVENSVAY	688	0.78
13	NSPGSASSVASQSIIAYTMS	679	0.78
13	NIDGYFKIYSKHTPINLVRG	196	0.78
14	DPSKPSKRSFIEDLLFNKVT	808	0.77
14	SVDCTMYICGDSTECSNLLL	735	0.77
14	SHPQFEKGGGSGGGGSGGSA	1261	0.77
15	VVNQNAQALNTLVKQLSSNF	951	0.76
15	KSFTVEKGIYQTSNFRVQPT	304	0.76
15	DCALDPLSETKCTLKSFTVE	290	0.76
15	LLKYNENGTITDAVDCALDP	276	0.76
15	YVRKDGEWVLLSTFLGRSLE	1223	0.76

© 2021. Bhattacharya M et al. Published online at http://www.aginganddisease.org/EN/10.14336/AD.2021.0517

15	SGINASVVNIQKEIDRLNEV	1170	0.76
16	ARDLICAQKFNGLTVLPPLL	846	0.75
16	RQIAPGQTGNIADYNYKLPD	408	0.75
16	ITRFQTLLALHISYLTPGDS	235	0.75
17	LTGTGVLTESNKKFLPFQQF	546	0.74
17	LYNSASFSTFKCYGVSPTKL	368	0.74
17	LIVNNATNVVIKVCEFQFCN	118	0.74
17	VVIGIVNNTVYDPLQPELDS	1128	0.74
18	LLHAPATVCGPKKSTNLVKN	517	0.73
18	YFPLQSYGFQPTYGVGYQPY	489	0.73
18	FSALEPLVDLPIGINITRFQ	220	0.73
18	KSWMESEFRVYSSANNCTFE	150	0.73
18	SQCVNFTTRTQLPPAYTNSF	13	0.73
18	PGHHHHHHHHSAWSHPQFEK	1248	0.73
19	CTQLNRALTGIAVEQDKNTQ	760	0.72
19	GSNVFQTRAGCLIGAEHVNN	639	0.72
19	WVLLSTFLGRSLEVLFQGPG	1230	0.72
19	FPREGVFVSNGTHWFVTQRN	1089	0.72
20	GLTVLPPLLTDEMIAQYTSA	857	0.71
20	TPTWRVYSTGSNVFQTRAGC	630	0.71
20	QPTYGVGYQPYRVVVLSFEL	498	0.71
20	GNIADYNYKLPDDFTGCVIA	416	0.71
20	GTITDAVDCALDPLSETKCT	283	0.71
20	SSGWTAGAAAYYVGYLQPRT	255	0.71
20	KEELDKYFKNHTSPDVDLGD	1149	0.71
21	SALGKLQDVVNQNAQALNTL	943	0.70
21	QIYKTPPIKDFGGFNFSQIL	787	0.70
21	SKVGGNYNYLYRLFRKSNLK	443	0.70
21	GRSLEVLFQGPGHHHHHHHH	1238	0.70
22	DEMIAQYTSALLAGTITSGW	867	0.69
22	QPTESIVRFPNITNLCPFGE	321	0.69
22	FKNHTSPDVDLGDISGINAS	1156	0.69
22	NTVYDPLQPELDSFKEELDK	1135	0.69
22	HWFVTQRNFYEPQIITTDNT	1101	0.69
23	GQSKRVDFCGKGYHLMSFPQ	1035	0.68
24	SNKKFLPFQQFGRDIADTTD	555	0.66
25	PKKSTNLVKNKCVNFNFNGL	527	0.65
25	QELGKYEQGSGYIPEAPRDG	1201	0.65
26	FNSAIGKIQDSLSSTASALG	927	0.64
26	WTFGAGAALQIPFAMQMAYR	886	0.64
26	SNQVAVLYQGVNCTEVPVAI	605	0.64
27	IDRLNEVAKNLNESLIDLQE	1183	0.63
28	IKDFGGFNFSQILPDPSKPS	794	0.61
28	TRTQLPPAYTNSFTRGVYYP	20	0.61
28	VPAQEKNFTTAPAICHDGKA	1068	0.61
29	EIYQAGSTPCNGVKGFNCYF	471	0.60
29	ICHDGKAHFPREGVFVSNGT	1081	0.60
30	AYSNNSIAIPTNFTISVTTE	706	0.59
31	ECDIPIGAGICASYQTQTNS	661	0.58
31	FPQSAPHGVVFLHVTYVPAQ	1052	0.58
32	KMSECVLGQSKRVDFCGKGY	1028	0.57
33	EIRASANLAATKMSECVLGQ	1017	0.56
34	IQDSLSSTASALGKLQDVVN	934	0.54

© 2021. Bhattacharya M et al. Published online at http://www.aginganddisease.org/EN/10.14336/AD.2021.0517

SUPPLEMENTARY DATA

Table S3. The B cell epitopes of the newly emerging variant (B.1.1.1.7) of SARS-CoV-2 S protein along with their length predicted by ABCPred server.

Rank	Sequence	Start position	Score
1	KVTLADAGFIKQYGDCLGDI	822	0.93
1	ADSFVIRGDEVRQIAPGQTG	394	0.93
1	PGDSSSGWTAGAAAYYVGYL	248	0.93
2	SLLIVNNATNVVIKVCEFQF	114	0.91
3	TKLNDLCFTNVYADSFVIRG	382	0.90
4	LNDILARLDKVEAEVQIDRL	974	0.88
4	SSNFGAISSVLNDILARLDK	964	0.88
4	IIAYTMSLGAENSVAYSNNS	689	0.88
4	VFNATRFASVYAWNRKRISN	338	0.88
5	QIDRLITGRLQSLQTYVTQQ	989	0.87
5	DCLGDIAARDLICAQKFNGL	836	0.87
6	VLTESNKKFLPFQQFGRDID	548	0.86
6	LVDLPIGINITRFQTLLALH	223	0.86
6	INLVRDLPQGFSALEPLVDL	207	0.86
6	EFVFKNIDGYFKIYSKHTPI	188	0.86
6	YVSQPFLMDLEGKQGNFKNL	167	0.86
6	RVYSSANNCTFEYVSQPFLM	155	0.86
6	VNNTVYDPLQPELDSFKEEL	1130	0.86
7	PSKRSFIEDLLFNKVTLADA	809	0.85
7	EVPVAIHADQLTPTWRVYST	616	0.85
7	RVVVLSFELLHAPATVCGPK	506	0.85
7	RSSVLHSTQDLFLPFFSNVT	44	0.85
8	RGVYYPDKVFRSSVLHSTQD	34	0.84
9	SVDCTMYICGDSTECSNLLL	732	0.83
9	TEILPVSMTKTSVDCTMYIC	721	0.83
9	TNLVKNKCVNFNFNGLTGTG	528	0.83
10	TLEILDITPCSFGGVSVITP	578	0.82
10	VNFNFNGLTGTGVLTESNKK	536	0.82
10	DPLSETKCTLKSFTVEKGIY	291	0.82
11	SNIIRGWIFGTTLDSKTQSL	96	0.81
11	TPPIKDFGGFNFSQILPDPS	788	0.81
11	NTQEVFAQVKQIYKTPPIKD	774	0.81
11	HVNNSYECDIPIGAGICASY	652	0.81
11	TRFQTLLALHRSYLTPGDSS	233	0.81
12	FAMQMAYRFNGIGVTQNVLY	895	0.80
12	RDIDDTTDAVRDPQTLEILD	564	0.80
12	HHHHHHSAWSHPQFEKGGGS	1249	0.80
13	TPGTNTSNQVAVLYQGVNCT	596	0.79
13	SVLYNSASFSTFKCYGVSPT	363	0.79
13	DKYFKNHTSPDVDLGDISGI	1150	0.79
14	TQLNRALTGIAVEQDKNTQE	758	0.78
14	TLDSKTQSLLIVNNATNVVI	107	0.78
15	AQYTSALLAGTITSGWTFGA	868	0.77
15	PLLTDEMIAQYTSALLAGTI	860	0.77
15	GGFNFSQILPDPSKPSKRSF	795	0.77
15	SNLLLQYGSFCTQLNRALTG	747	0.77
15	LQSYGFQPTYGVGYQPYRVV	489	0.77
15	PFERDISTEIYQAGSTPCNG	460	0.77
15	VEKGIYQTSNFRVQPTESIV	305	0.77
16	NQKLIANQFNSAIGKIQDSL	916	0.76
16	TFGAGAALQIPFAMQMAYRF	884	0.76
16	NSIAIPINFTISVTTEILPV	707	0.76

© 2021. Bhattacharya M et al. Published online at http://www.aginganddisease.org/EN/10.14336/AD.2021.0517

16	ISGTNGTKRFDNPVLPFNDG	68	0.76
16	LPDDFTGCVIAWNSNNLDSK	422	0.76
16	DISGINASVVNIQKEIDRLN	1165	0.76
17	QKFNGLTVLPPLLTDEMIAQ	850	0.75
17	AENSVAYSNNSIAIPINFTI	698	0.75
17	HAPATVCGPKKSTNLVKNKC	516	0.75
17	VQPTESIVRFPNITNLCPFG	317	0.75
17	KQGNFKNLREFVFKNIDGYF	179	0.75
17	SQCVNLTTRTQLPPAYTNSF	13	0.75
17	YEPQIITTHNTFVSGNCDVV	1107	0.75
17	SNGTHWFVTQRNFYEPQIIT	1094	0.75
18	KCYGVSPTKLNDLCFTNVYA	375	0.74
18	QPRTFLLKYNENGTITDAVD	268	0.74
18	QGSGYIPEAPRDGQAYVRKD	1205	0.74
19	KRISNCVADYSVLYNSASFS	353	0.73
19	AICHDGKAHFPREGVFVSNG	1077	0.73
20	ALNTLVKQLSSNFGAISSVL	955	0.72
20	NYLYRLFRKSNLKPFERDIS	447	0.72
20	PGQTGKIADYNYKLPDDFTG	409	0.72
20	LSTFLGRSLEVLFQGPGHHH	1230	0.72
21	FDNPVLPFNDGVYFASTEKS	77	0.71
21	RVYSTGSNVFQTRAGCLIGA	631	0.71
21	DAVDCALDPLSETKCTLKSF	284	0.71
21	TFVSGNCDVVIGIVNNTVYD	1117	0.71
21	CVLGQSKRVDFCGKGYHLMS	1029	0.71
22	FQFCNDPFLGVYHKNNKSWM	131	0.70
22	SLEVLFQGPGHHHHHHHHSA	1237	0.70
22	VPAQEKNFTTAPAICHDGKA	1065	0.70
23	TEIYQAGSTPCNGVEGFNCY	467	0.69
23	PQSAPHGVVFLHVTYVPAQE	1050	0.69
23	CGKGYHLMSFPQSAPHGVVF	1040	0.69
24	TAGAAAYYVGYLQPRTFLLK	256	0.68
24	QQLIRAAEIRASANLAATKM	1007	0.68
25	PIGAGICASYQTQTNSHGSA	662	0.67
25	QTRAGCLIGAEHVNNSYECD	641	0.67
26	SALGKLQDVVNQNAQALNTL	940	0.66
26	FLPFQQFGRDIDDTTDAVRD	556	0.66
27	AWNSNNLDSKVGGNYNYLYR	432	0.65
28	KYNENGTITDAVDCALDPLS	275	0.64
28	AWSHPQFEKGGGSGGGGSGG	1256	0.64
28	QKEIDRLNEVAKNLNESLID	1177	0.64
29	PNITNLCPFGEVFNATRFAS	327	0.61
30	QSLQTYVTQQLIRAAEIRAS	999	0.60
31	NEVAKNLNESLIDLQELGKY	1184	0.59
32	NSAIGKIQDSLSSTASALGK	925	0.57
32	VFLHVTYVPAQEKNFTTAPA	1058	0.57
32	SANLAATKMSECVLGQSKRV	1018	0.57
33	DSLSSTASALGKLQDVVNQN	933	0.54
34	STPCNGVEGFNCYFPLQSYG	474	0.52

© 2021. Bhattacharya M et al. Published online at http://www.aginganddisease.org/EN/10.14336/AD.2021.0517

SUPPLEMENTARY DATA

Table S4. The B cell epitopes of the newly emerging variant (B.1.1.28) of SARS-CoV-2 S protein along with their length predicted by ABCPred server.

Rank	Sequence	Start position	Score
1	VTLADAGFIKQYGDCLGDIA	826	0.90
1	IGAEHVNNSYECDIPIGAGI	651	0.90
1	TNVYADSFVIRGDEVRQIAP	393	0.90
1	VYYHKNNKSWMESEFRVYSS	143	0.90
2	IHVSGTNGTKRFDNPVLPFN	68	0.89
2	SYLTPGDSSSGWTAGAAAYY	247	0.89
3	KHTPINLVRDLPQGFSALEP	206	0.88
3	VVIKVCEFQFCNDPFLGVYY	126	0.88
4	DRLITGRLQSLQTYVTQQLI	994	0.87
4	GVNCTEVPVAIHADQLTPTW	614	0.87
4	PCSFGGVSVITPGTNTSNQV	589	0.87
4	PFGEVFNATRFASVYAWNRK	337	0.87
4	VTQQLIRAAEIRASANLAAT	1008	0.87
5	DNTFVSGNCDVVIGIVNNTV	1118	0.86
5	TTLDSKTQSLLIVNNATNVV	108	0.86
6	KQYGDCLGDIAARDLICAQK	835	0.85
6	RSSVLHSTQDLFLPFFSNVT	44	0.85
6	KRISNCVADYSVLYNSASFS	356	0.85
6	APRDGQAYVRKDGEWVLLST	1216	0.85
7	NVLYENQKLIANQFNSAIGK	914	0.84
7	RGVYYPDKVFRSSVLHSTQD	34	0.84
8	ISSVLNDILSRLDKVEAEVQ	973	0.83
8	AGTITSGWTFGAGAALQIPF	879	0.83
8	ISVTTEILPVSMTKTSVDCT	720	0.83
8	PVAIHADQLTPTWRVYSTGS	621	0.83
9	QDKNTQEVFAQVKQIYKTPP	774	0.82
9	QQFGRDIADTTDAVRDPQTL	563	0.82
9	KNKCVNFNFNGLTGTGVLTE	535	0.82
9	SNLKPFERDISTEIYQAGST	459	0.82
9	DCALDPLSETKCTLKSFTVE	290	0.82
9	DLEGKQGNFKNLREFVFKNI	178	0.82
9	VSQPFLMDLEGKQGNFKNLR	171	0.82
9	YSSANNCTFEYVSQPFLMDL	160	0.82
10	YQPYRVVVLSFELLHAPATV	505	0.81
10	GCVIAWNSNNLDSKVGGNYN	431	0.81
11	ECSNLLLQYGSFCTQLNRAL	748	0.80
11	GVSPTKLNDLCFTNVYADSF	381	0.80
12	LVKQLSSNFGAISSVLNDIL	962	0.79
12	GVYFASTEKSNIIRGWIFGT	89	0.79
12	CGKGYHLMSFPQSAPHGVVF	1043	0.79
13	ASQSIIAYTMSLGAENSVAY	688	0.78
13	NSPGSASSVASQSIIAYTMS	679	0.78
13	NIDGYFKIYSKHTPINLVRD	196	0.78
14	DPSKPSKRSFIEDLLFNKVT	808	0.77
14	SVDCTMYICGDSTECSNLLL	735	0.77
14	SHPQFEKGGGSGGGGSGGSA	1261	0.77
15	VVNQNAQALNTLVKQLSSNF	951	0.76
15	LLKYNENGTITDAVDCALDP	276	0.76
15	YVRKDGEWVLLSTFLGRSLE	1223	0.76
15	SGINASVVNIQKEIDRLNEV	1170	0.76
16	ARDLICAQKFNGLTVLPPLL	846	0.75
16	RQIAPGQTGNIADYNYKLPD	408	0.75

© 2021. Bhattacharya M et al. Published online at http://www.aginganddisease.org/EN/10.14336/AD.2021.0517

SUPPLEMENTARY DATA

16	ITRFQTLLALHRSYLTPGDS	235	0.75
16	SQCVNLTTRTQLPPAYTNSF	13	0.75
17	LTGTGVLTESNKKFLPFQQF	546	0.74
17	LYNSASFSTFKCYGVSPTKL	368	0.74
17	KSFTVEKGIYQTSNFRVQPT	304	0.74
17	LIVNNATNVVIKVCEFQFCN	118	0.74
17	VVIGIVNNTVYDPLQPELDS	1128	0.74
18	LLHAPATVCGPKKSTNLVKN	517	0.73
18	YFPLQSYGFQPTYGVGYQPY	489	0.73
18	FSALEPLVDLPIGINITRFQ	220	0.73
18	KSWMESEFRVYSSANNCTFE	150	0.73
18	PGHHHHHHHHSAWSHPQFEK	1248	0.73
19	CTQLNRALTGIAVEQDKNTQ	760	0.72
19	GSNVFQTRAGCLIGAEHVNN	639	0.72
19	WVLLSTFLGRSLEVLFQGPG	1230	0.72
19	FPREGVFVSNGTHWFVTQRN	1089	0.72
20	TPTWRVYSTGSNVFQTRAGC	630	0.71
20	QPTYGVGYQPYRVVVLSFEL	498	0.71
20	GNIADYNYKLPDDFTGCVIA	416	0.71
20	GTITDAVDCALDPLSETKCT	283	0.71
20	KEELDKYFKNHTSPDVDLGD	1149	0.71
21	SALGKLQDVVNQNAQALNTL	943	0.70
21	QIYKTPPIKDFGGFNFSQIL	787	0.70
21	SKVGGNYNYLYRLFRKSNLK	443	0.70
21	GRSLEVLFQGPGHHHHHHHH	1238	0.70
22	QPTESIVRFPNITNLCPFGE	321	0.69
22	FKNHTSPDVDLGDISGINAS	1156	0.69
22	NTVYDPLQPELDSFKEELDK	1135	0.69
22	HWFVTQRNFYEPQIITTDNT	1101	0.69
23	SSGWTAGAAAYYVGYLQPRT	255	0.68
23	GQSKRVDFCGKGYHLMSFPQ	1035	0.68
24	SNKKFLPFQQFGRDIADTTD	555	0.66
25	PKKSTNLVKNKCVNFNFNGL	527	0.65
25	QELGKYEQGSGYIPEAPRDG	1201	0.65
26	FNSAIGKIQDSLSSTASALG	927	0.64
26	WTFGAGAALQIPFAMQMAYR	886	0.64
26	SNQVAVLYQGVNCTEVPVAI	605	0.64
27	IDRLNEVAKNLNESLIDLQE	1183	0.63
28	IKDFGGFNFSQILPDPSKPS	794	0.61
28	VPAQEKNFTTAPAICHDGKA	1068	0.61
29	GLTVLPPLLTDEMIAQYTSA	706	0.59
29	EIYQAGSTPCNGVKGFNCYF	857	0.60
29	ICHDGKAHFPREGVFVSNGT	471	0.60
30	AYSNNSIAIPTNFTISVTTE	1081	0.60
31	ECDIPIGAGICASYQTQTNS	661	0.58
31	FPQSAPHGVVFLHVTYVPAQ	1052	0.58
32	KMSECVLGQSKRVDFCGKGY	1028	0.57
33	EIRASANLAATKMSECVLGQ	1017	0.56
34	IQDSLSSTASALGKLQDVVN	934	0.54

© 2021. Bhattacharya M et al. Published online at http://www.aginganddisease.org/EN/10.14336/AD.2021.0517
